# Duration of palliative care before death in international routine practice: a systematic review and meta-analysis

**DOI:** 10.1186/s12916-020-01829-x

**Published:** 2020-11-26

**Authors:** Roberta I. Jordan, Matthew J. Allsop, Yousuf ElMokhallalati, Catriona E. Jackson, Helen L. Edwards, Emma J. Chapman, Luc Deliens, Michael I. Bennett

**Affiliations:** 1 Academic Unit of Palliative Care, Leeds Institute of Health Sciences, University of Leeds, Leeds, UK; 2 Wolfson Palliative Care Research Centre, Hull York Medical School, University of Hull, Hull, UK; 3 End-of-Life Care Research Group, Ghent University, Ghent, Belgium; 4 Vrije Universiteit Brussel, Brussels, Belgium

**Keywords:** Palliative care, Health services accessibility, Hospice care, Duration of care, Systematic review

## Abstract

**Background:**

Early provision of palliative care, at least 3–4 months before death, can improve patient quality of life and reduce burdensome treatments and financial costs. However, there is wide variation in the duration of palliative care received before death reported across the research literature. This study aims to determine the duration of time from initiation of palliative care to death for adults receiving palliative care across the international literature.

**Methods:**

We conducted a systematic review and meta-analysis that was registered with PROSPERO (CRD42018094718). Six databases were searched for articles published between Jan 1, 2013, and Dec 31, 2018: MEDLINE, Embase, CINAHL, Global Health, Web of Science and The Cochrane Library, as well undertaking citation list searches. Following PRISMA guidelines, articles were screened using inclusion (any study design reporting duration from initiation to death in adults palliative care services) and exclusion (paediatric/non-English language studies, trials influencing the timing of palliative care) criteria. Quality appraisal was completed using Hawker’s criteria and the main outcome was the duration of palliative care (median/mean days from initiation to death).

**Results:**

One hundred sixty-nine studies from 23 countries were included, involving 11,996,479 patients. Prior to death, the median duration from initiation of palliative care to death was 18.9 days (IQR 0.1), weighted by the number of participants. Significant differences between duration were found by disease type (15 days for cancer vs 6 days for non-cancer conditions), service type (19 days for specialist palliative care unit, 20 days for community/home care, and 6 days for general hospital ward) and development index of countries (18.91 days for very high development vs 34 days for all other levels of development). Forty-three per cent of studies were rated as ‘good’ quality. Limitations include a preponderance of data from high-income countries, with unclear implications for low- and middle-income countries.

**Conclusions:**

Duration of palliative care is much shorter than the 3–4 months of input by a multidisciplinary team necessary in order for the full benefits of palliative care to be realised. Furthermore, the findings highlight inequity in access across patient, service and country characteristics. We welcome more consistent terminology and methodology in the assessment of duration of palliative care from all countries, alongside increased reporting from less-developed settings, to inform benchmarking, service evaluation and quality improvement.

## Background

Palliative care aims to improve the quality of life of patients with life-limiting illnesses through prevention and relief of suffering [1]. Recent systematic reviews and meta-analyses demonstrate that early integration of specialist palliative care can improve quality of life for patients with advanced, incurable illness, including reducing symptom intensity, hospitalisation, aggressive treatments and associated costs at the end of life [2–4]. Studies included patients with malignant and non-malignant disease. Definitions of early integration vary. Although there is a paucity of well-conducted randomised controlled trials (RCTs) with mixed findings across trials, evidence suggests that ‘for full benefits of palliative care to be realized, continuity by a multidisciplinary team is needed for at least 3–4 months’ [5] to realise maximum benefit.

The 2018 *Lancet* Commission on Palliative Care and Pain Relief identified a lack of palliative care and pain relief globally [6]. By 2060, the burden of serious health-related suffering is expected to increase almost twofold, most rapidly in low-income countries [7]. Studies reporting on the duration of palliative care vary (e.g. from a median of 18–57 days and mean of 30–70 days), and suggest that early integration is not routine practice [8–19].

To date, there have been no attempts to systematically summarise reports on the duration of palliative care across the research literature. Doing so with a global focus could allow local and national services to benchmark using an international standard, determine country variations reflecting differences in wealth and palliative care development, and would identify the gap between current routine practice and the ideal duration of 3–4 months of palliative care.

This systematic review aims to identify studies reporting on the time interval between initiation of specialised palliative care services and death for adult patients within routine clinical practice and to explore associated patient, service and country characteristics which influence this duration.

## Methods

We conducted a systematic review and meta-analysis. The study protocol was registered with PROSPERO (CRD42018094718) on 30 April 2018. Ethics approval was not required for this secondary data analysis.

### Data sources and searches

Databases searched included MEDLINE (1946 to January 2019), Embase (1947 to January 2019), CINAHL (1960 to January 2019), Global Health (1973 to January 2019), Web of Science (1990 to January 2019) and The Cochrane Library. We conducted database searches on 2 October 2017 with additional updates on 12 February 2018 and 15 January 2019. The search strategy included terms for palliative care, duration of palliative care and referral (see example MEDLINE strategy provided in Additional file 1: Fig S1). Additional studies were identified through hand-searching of reference and citation lists of included studies. Search strategies (e.g. Additional file 1: Fig S1) were used to conduct a scoping search in October 2020 to determine whether any eligible population-based or large-scale studies had been published since January 2019. Of the 667 abstracts identified, no eligible population-based or large studies were identified so an updated extraction and analysis including data from 2019 was not undertaken.

### Study selection

Studies were included if they reported on adult patients (≥ 18 years old) referred to or admitted under adult palliative care services. Palliative care services were defined as healthcare services which either self-define as specialist palliative care services or solely or majorly practise palliative care. These services could be in any setting including general hospital wards, specialist inpatient units/hospices or community settings (outpatients/day units/home care). Studies were only included if they reported length-of-stay in a specialist inpatient palliative care unit, referral-to-death time interval in any setting or survival time after palliative care referral. We limited search results to studies published from 1 January 2013 to 31 December 2018 to reflect contemporary practice. Included studies could be of any study design. Unpublished study data were included after contact with study investigators. Studies were excluded if they included children (< 18 years old) or referrals to paediatric palliative care services and did not report adult data separately, reported on referrals solely to bereavement services or non-palliative care services, were randomised trials where the intervention influenced the timing of palliative care or were not in the English language.

### Data extraction and quality assessment

Three authors (RIJ/CEJ/HLE) independently screened 10% of the titles and abstracts of studies identified. Discrepancies in screening inclusion and exclusion were resolved through discussion, and then review by a fourth author (MJA). As concordance between authors was greater than 90%, each author screened a portion of the remaining abstracts alone (assigned randomly and evenly distributed). Full-text articles were assessed for eligibility and, if included, data extraction was performed using a piloted form by three independent authors (RIJ/YE/HLE).

Data were extracted from each study on the following sample and methodological characteristics: country of origin, country level of human development according to the UNDP Development Index, country palliative care development level according to the WHPCA categorisation of palliative care development, study design, number of patients, percentage of patients alive at the end of the study period, summaries of age, gender and ethnicity (% White Caucasian) of study participants, each cohort’s type of disease, type of palliative care service referred to, the geographical level of analysis for each study (local, regional, national, international), the statistical summary of duration in days (mean, median or both) and terminology used to describe the duration of palliative care (survival, referral-to-death, length of stay). We extracted data on the primary outcome of the duration of palliative care before death in days (median or mean). Included study authors were contacted for missing data. Study quality of individual studies was assessed using Hawker’s criteria for reviewing disparate observational data systematically [20]. The criteria consist of 9 items, each with a score of 1–4 (total score of 36). We rated studies with scores of ≤ 18 as poor, 19–27 as fair and > 27 as good, consistent with Boer et al.’s approach [21]. Four authors piloted the criteria with 10% of the included studies, with concordance of > 90% achieved prior to individual allocation of remaining studies (RIJ/MJA/YE/EJC).

### Data synthesis and analysis

A meta-analysis was performed to synthesise reporting of the median number of days palliative care was initiated prior to death. We used the median as the preferred measure of central tendency as it is less affected by outliers and better reflects skewed data. The number of days from palliative care initiation to death was reported across the included articles in one of three ways: as a median, as a mean or including both a median and mean value. For studies reporting mean values only, linear regression modelling was used to derive a median value (see Additional file 1: Fig S2). This was calculated through examining the relationship between mean and median values in articles where both values were reported. The trend line was then applied to derive median values where included studies only reported a mean number of days from palliative care initiation to death. We then weighted median values for each study according to the number of study participants it contained. We then combined median values from all studies to calculate a final weighted median value and the interquartile range (IQR) to summarise duration of palliative care.

Additional analyses were performed to investigate the impact of country level of human development (United Nations Development Programme Human Development Index categories of very high, high, medium, low, other), country level of palliative care development (Worldwide Hospice Palliative Care Alliance 2011 categorisation of palliative care development using 1, 2, 3a, 3b, 4a, 4b), type of disease (malignant, non-malignant, mixed) and type of setting of the palliative care service (specialist palliative care unit, community/home, combined, general hospital ward, unspecified) on the duration of palliative care. We used the Mann-Whitney *U* test to compare the distribution of medians using a significance level of *p* = 0.05.

Sensitivity analyses were performed to assess the robustness of the primary outcome. Sensitive analyses were undertaken to determine the impact of the characteristics of included studies on the overall weighted median duration of palliative care derived from all studies. A number of characteristics were identified for sensitivity analysis. These included studies where the mean duration of palliative care had been converted to a median value, studies with small sample sizes (< 100 participants), studies analysing local and regional data (defined as ≥ 1 centres in the same geographical region within one country), studies reporting > 5% survival at the end of the study period, studies reporting length-of-stay in palliative care units and studies with poor/fair ratings for methodological quality. For each characteristic included in the sensitivity analyses, we identified all studies with the characteristic of interest (e.g. studies with small sample sizes < 100 participants), removed data from all studies with the characteristic, and then recalculated the final weighted median value with interquartile range (IQR) and median absolute deviation (MAD) to summarise duration of palliative care less the data from studies with the characteristic being explored. This enabled us to explore the influence of characteristics of interest on the overall weighted median duration of palliative care. We also conducted a post hoc analysis excluding the USA (United States of America) studies, given differences found between studies from and outside of the USA.

We used IBM SPSS Statistics 22 for data analysis. Reporting is aligned with the PRISMA checklist for reporting systematic reviews and meta-analyses (see Additional file 2: Table S1).

## Results

**Fig. 1:**
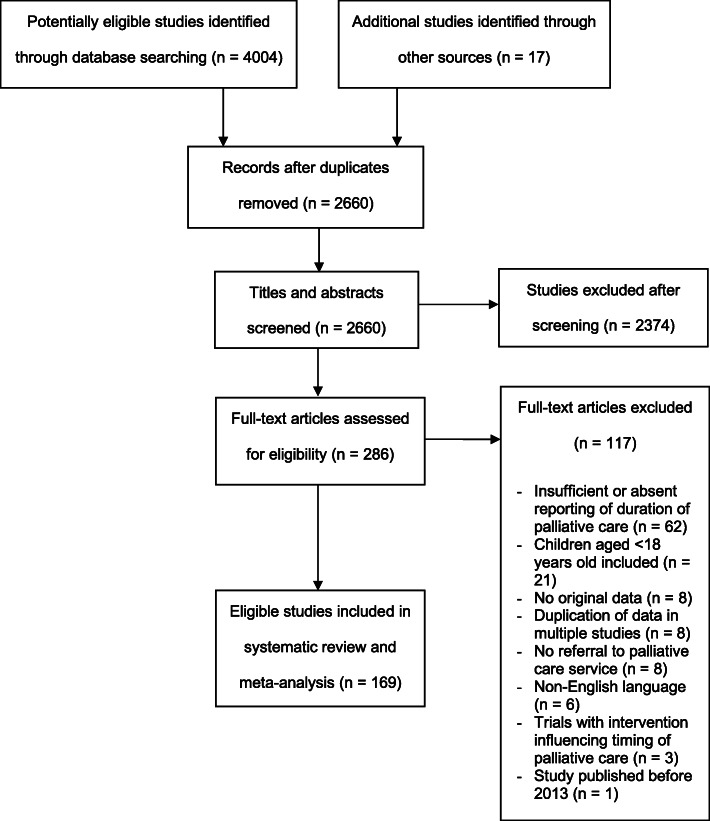
PRISMA flowchart outlining article selection

Two thousand six hundred sixty studies were screened, with 169 studies included in the systematic review and meta-analysis. Studies were excluded at the screening stage as their titles/abstracts did not meet the inclusion criteria broadly. Reasons for exclusion at the final eligibility stage are outlined in Fig. 1.

Table 1 summarises the individual characteristics of the included studies. All included studies were used in the meta-analysis.

**Table 1:** Overview of the key characteristics of articles included the review

Study author and citation	Country	UNDP HDI	WHPCA palliative care development	Study design	No. of patients	% patients alive at end of study period	Age	Gender (% female)	Ethnicity (% White Caucasian)	Type of disease	Type of palliative care service	Level of analysis	Duration of palliative care (days)	Statistical summary reported	Terminology used to report duration of palliative care	Hawker’s criteria rating (score)
Aeckerle, 2013 [22]	Germany	Very high	4b	RO	225	9%	66*	100%	U	Malignant	SPCU	Local	59*	Median	Survival	Fair (26)
Alsirafy, 2013 [23]	Egypt	Medium	3a	RO	123	0%	53*	57%	U	Malignant	SPCU	Local	66*	Median	Survival	Fair (23)
Bakitas, 2013 [24]	USA	Very high	4b	RO	132	13%	76*	47%	98%	Non-malignant	Combined	Local	21*	Median	Referral-to-death	Fair (26)
Beernaert, 2013 [25]	Belgium	Very high	4b	RO	577	0%	U	51%	U	Mixed	Combined	National	17.95*^†^	Median	Referral-to-death	Fair (27)
Cheung, 2013 [26]	USA	Very high	4b	RO	5420	0%	82^‡^	72%	95%	Mixed	SPCU	National	17.67*^†^	Median	Referral-to-death	Good (29)
Corbett, 2013 [27]	Australia	Very high	4b	PO	276	46%	61^‡^	42%	U	Malignant	Combined	Local	34*	Median	Referral-to-death	Fair (22)
D’Angelo, 2013 [28]	Italy	Very high	4b	RO	5320	9%	74^‡^	50%	U	Malignant	Combined	Local	21*, 38.8^‡^	Median and mean	Survival	Fair (27)
Dong, 2013 [29]	USA	Very high	4b	PO	1423	17%	73^‡§^	60%	U	Mixed	SPCU	Regional	95.44^‡†^	Mean	Referral-to-death	Good (28)
Eti, 2013 [30]	USA	Very high	4b	RO	1837	73%	70*	60%	U	Mixed	SPCU	Local	4*	Median	Length-of-stay	Fair (24)
Gerber, 2013 [31]	USA	Very high	4b	PO	48	U	U	U	U	Unspecified	SPCU	Local	7*	Median	Length-of-stay	Poor (15)
Harris, 2013 [32]	USA	Very high	4b	RO	8669	0%	75^‡^	58%	U	Mixed	SPCU	Regional	31*	Median	Length-of-stay	Fair (24)
Hussain, 2013 [33]	UK	Very high	4b	RO	62	0%	67^‡^	50%	100%	Non-malignant	Community/home	Local	336*	Median	Referral-to-death	Good (32)
Kelley, 2013 [34]	USA	Very high	4b	RO	1064	0%	84^‡^	54%	85%	Mixed	SPCU	National	49*	Median	Length-of-stay	Fair (26)
Mack, 2013 [35]	USA	Very high	4b	RO	24,226	≤ 10%	U	48%	U	Malignant	Combined	National	15.53*^†^, 45.08^‡†^	Median and mean	Referral-to-death	Good (28)
Meng, 2013 [36]	USA	Very high	4b	RO	136	0%	87^‡^	78%	70%	Mixed	SPCU	Regional	15*, 47.9^‡^	Median and mean	Length-of-stay	Good (29)
Mercadante, 2013 [37]	Italy	Very high	4b	PO	236	14%	73^‡^	50%	U	Malignant	SPCU	Regional	18.4^‡^	Mean	Length-of-stay	Fair (26)
Nabal, 2013 [38]	Spain	Very high	4a	PO	698	26%	74^‡^	40%	U	Malignant	Community/home	Regional	65^‡^	Mean	Referral-to-death	Good (28)
Nevadunsky, 2013 [39]	USA	Very high	4b	RO	49	0%	61^‡^	U	35%	Malignant	Combined	Local	16*	Median	Referral-to-death	Fair (26)
Pattenden, 2013 [40]	UK	Very high	4b	PO	80	6%	81^‡^	39%	91%	Non-malignant	Community/home	National	41.41*^†^	Median	Survival	Good (29)
Redahan, 2013 [41]	Ireland	Very high	4b	RO	48	0%	64^‡^	33%	U	Non-malignant	Unspecified	Local	12*	Median	Referral-to-death	Fair (26)
Sengupta, 2013 [42]	USA	Very high	4b	RO	4375	U	U	45%	91%	Mixed	Combined	National	16.2*, 65.3^‡^	Median and mean	Referral-to-death	Fair (19)
Speer, 2013 [43]	USA	Very high	4b	RO	279	0%	81*	56%	34%	Mixed	Community/home	Local	16*	Median	Length-of-stay	Fair (22)
Wallace, 2013 [44]	Ireland	Very high	4b	RO	92	22%	77^‡^	65%	U	Non-malignant	General hospital ward	Local	4.6^‡^	Mean	Referral-to-death	Good (28)
Weckmann, 2013 [45]	USA	Very high	4b	RO	98	0%	U	U	U	Mixed	General hospital ward	Local	13.16^‡^	Mean	Length-of-stay	Fair (21)
Zdenkowski, 2013 [46]	Australia	Very high	4b	RO	550	33%^§^	64^‡d^	50%^d^	U	Malignant	Combined	Regional	77*	Median	Referral-to-death	Fair (27)
Zheng, 2013 [47]	USA	Very high	4b	RO	817	0%	87^‡^	70%	89%	Mixed	Community/home	Regional	23.5*, 93^‡^	Median and mean	Length-of-stay	Fair (26)
Bogasky, 2014 [48]	USA	Very high	4b	RO	3,008,137	17%	U	58%	U	Mixed	Combined	National	19*, 83^‡^	Median and mean	Referral-to-death	Fair (25)
Brown, 2014 [49]	USA	Very high	4b	RO	144	0%	60*^§^	100%	68%^d^	Malignant	Combined	Local	35.5*	Median	Referral-to-death	Good (30)
Casarett, 2014 [50]	USA	Very high	4b	RO	49,370	16%	79^‡^	56%	89%	Mixed	SPCU	Regional	24.74^‡†^	Mean	Length-of-stay	Good (29)
Chai, 2014 [51]	Canada	Very high	4b	PO	129	0%	71^‡^	55%	U	Malignant	Community/home	Local	92*, 145.85^‡^	Median and mean	Referral-to-death	Fair (27)
Eastman, 2014 [52]	Australia	Very high	4b	RO	6004	35%	U	U	U	Unspecified	SPCU	Regional	12.8^‡^	Mean	Length-of-stay	Fair (24)
Fullerton, 2014 [53]	Australia	Very high	4b	RO	38	11%	U	U	U	Unspecified	SPCU	Local	15.1^‡^	Mean	Length-of-stay	Poor (13)
Guay, 2014 [54]	USA	Very high	4b	RO	435	U	57*	54%	62%	Malignant	Community/home	Local	214.91*^†^	Median	Survival	Good (29)
Hui, 2014 [55]	USA	Very high	4b	RO	368	0%	60^‡^	52%	62%	Malignant	Combined	Local	62.33*^†^	Median	Referral-to-death	Good (31)
Kang, 2014 [56]	Taiwan	Very high	4a	RO	2154	32%	74^‡^	49%	U	Mixed	SPCU	National	10.41^‡†^	Mean	Length-of-stay	Fair (26)
Kao, 2014 [57]	Taiwan	Very high	4a	RO	2020	15%	58*^†^	41%	U	Malignant	General hospital ward	Regional	33.71*^†^	Median	Referral-to-death	Good (29)
Keim-Malpass, 2014 [58]	USA	Very high	4b	RO	30	0%	31^‡§^	51%^§^	79%^§^	Malignant	General hospital ward	Local	12.8^‡^	Mean	Referral-to-death	Good (29)
Koivu, 2014 [59]	Finland	Very high	4a	RO	138	0%	75*	64%	U	Malignant	SPCU	Regional	32^‡^	Mean	Length-of-stay	Fair (23)
Obermeyer, 2014 [60]	USA	Very high	4b	RO	18,165	0%	80^‡^	52%	88%	Malignant	Combined	National	11*	Median	Length-of-stay	Fair (26)
Olmsted, 2014 [61]	USA	Very high	4b	RO	77,267	0%	U	2%^§^	71%^§^	Mixed	Combined	National	23.63*^†^	Median	Referral-to-death	Good (29)
Scheffey, 2014 [62]	USA	Very high	4b	RO	1368	0%	U	U	U	Mixed	SPCU	Regional	15*	Median	Length-of-stay	Fair (25)
Seow, 2014 [63]	Canada	Very high	4b	RO	3912	0%	75*	52%	U	Mixed	Community/home	Regional	73^‡^	Mean	Length-of-stay	Fair (26)
Sexauer, 2014 [64]	USA	Very high	4b	RO	53	0%	64^‡^	51%	U	Malignant	Combined	Local	7.98*^†^	Median	Length-of-stay	Fair (27)
Shin, 2014 [65]	USA	Very high	4b	RO	610	20%	59^‡^	47%	66%	Malignant	SPCU	Local	32.53*^†^, 8^‡^	Median and mean	Survival	Good (29)
Unroe, 2014 [66]	USA	Very high	4b	RO	3771	17%	79^‡^	59%	62%	Mixed	Combined	National	22*, 84.5^‡^	Median and mean	Referral-to-death	Poor (17)
Wachterman, 2014 [67]	USA	Very high	4b	RO	1415	16%	75^‡^	0%	82%	Mixed	Combined	National	16.71*^†^	Median	Referral-to-death	Good (29)
Yamagishi, 2014 [68]	Japan	Very high	4b	RO	693	0%	72^‡^	40%	U	Malignant	Community/home	National	35*	Median	Referral-to-death	Good (29)
Yeung, 2014 [69]	USA	Very high	4b	RO	6982	0%	U	49%	89%	Malignant	SPCU	National	13*	Median	Length-of-stay	Good (28)
Alsirafy, 2015 [70]	Saudi Arabia	Very high	3a	RO	328	0%	U	50%	U	Malignant	Combined	Local	16.1^‡^	Mean	Length-of-stay	Fair (23)
Chiang, 2015 [71]	Taiwan	Very high	4a	RO	566	0%	69^‡^	39%	U	Malignant	Combined	National	23*, 54.7^‡^	Median and mean	Referral-to-death	Good (30)
Choi, 2015 [72]	South Korea	Very high	3a	RO	84	0%	64* ^§^	35%^§^	U	Malignant	SPCU	Local	12.33*^†^, 26.25^‡†^	Median and mean	Length-of-stay	Fair (24)
Colman, 2015 [73]	Canada	Very high	4b	RO	24	0%	57^‡^	46%	U	Mixed	Combined	Local	14*	Median	Referral-to-death	Fair (25)
Dingfield, 2015 [74]	USA	Very high	4b	RO	125,634	3%	78^‡^	56%	87%	Mixed	SPCU	National	53^‡^	Mean	Length-of-stay	Good (30)
Dougherty, 2015 [75]	USA	Very high	4b	RO	85,581	14%	74^‡^	52%	88%	Mixed	SPCU	National	79.57^‡†^	Mean	Referral-to-death	Fair (26)
El-Jawahri, 2015 [76]	USA	Very high	4b	RO	49	0%	70^‡§^	41%^§^	97%^§^	Malignant	Combined	Regional	7*	Median	Referral-to-death	Fair (26)
Gage, 2015 [77]	UK	Very high	4b	RO	688	0%	75^‡^	44%	U	Unspecified	Combined	Regional	70.54^‡†^	Mean	Referral-to-death	Good (30)
Gozalo, 2015 [78]	USA	Very high	4b	RO	261,252	U	85^‡§^	65%^§^	U	Mixed	SPCU	National	19.20*^†^, 83.35^‡†^	Median and mean	Length-of-stay	Fair (22)
Gu, 2015 [79]	China	High	4a	RO	244	0%	63^‡^	49%	U	Malignant	SPCU	Local	19*, 23.54^‡^	Median and mean	Survival	Good (29)
Gupte, 2015 [80]	USA	Very high	4b	RO	1801	U	72^‡^	49%	93%	Malignant	Combined	National	21.81*^†^, 68.66^‡†^	Median and mean	Length-of-stay	Good (32)
Hennemann-Krause, 2015 [81]	Brazil	High	3a	PO	12	0%	68^‡^	42%	U	Malignant	Community/home	Local	195^‡^	Mean	Referral-to-death	Good (28)
Hui, 2015 [82]	Multicentre - USA and Brazil	Very high & High	4b & 3a	PO	357	43%	58^‡^	55%	U	Malignant	SPCU	Inter-national	6*	Median	Length-of-stay	Good (31)
Kao, 2015 [83]	Taiwan	Very high	4a	RO	462	0%	68^‡^	30%	U	Malignant	Combined	National	19*, 36.37^‡^	Median and mean	Referral-to-death	Good (31)
Kim, 2015 [84]	South Korea	Very high	3a	RO	198	0%	66^‡^	41%	U	Malignant	SPCU	Local	18*	Median	Length-of-stay	Good (28)
Kozlov, 2015 [85]	USA	Very high	4b	RO	160	13%	U	U	U	Mixed	General hospital ward	Local	23*, 73.89^‡^	Median and mean	Referral-to-death	Fair (21)
Lee, 2015 [86]	South Korea	Very high	3a	RO	609	0%	U	52%	U	Malignant	SPCU	Local	21*	Median	Survival	Fair (24)
Myers, 2015 [87]	Canada	Very high	4b	PO	368	0%	65^‡^	56%	U	Malignant	Community/home	Local	121*, 171.7^‡^	Median and mean	Survival	Fair (21)
O’Connor, 2015 [88]	USA	Very high	4b	RO	122	0%	55^‡^	100%	76%	Malignant	Combined	Local	17*	Median	Referral-to-death	Good (29)
Pineau, 2015 [89]	Canada	Very high	4b	RO	28	0%	U	U	U	Unspecified	SPCU	Local	120^‡^	Mean	Length-of-stay	Poor (13)
Zakhour, 2015 [90]	USA	Very high	4b	RO	55	0%	70^‡§^	100%	79%^§^	Malignant	Combined	Local	28*	Median	Referral-to-death	Good (28)
Bauman, 2016 [91]	USA	Very high	4b	RO	46	0%	64*^§^	68%	77%	Malignant	Combined	Local	96*	Median	Referral-to-death	Fair (26)
Bennett, 2016 [10]	UK	Very high	4b	RO	4650	0%	75*	49%	U	Mixed	Combined	Regional	34*	Median	Referral-to-death	Good (29)
Brooks, 2016 [92]	USA	Very high	4b	RO	650	0%	U	39%	73%	Malignant	Combined	National	21.44*	Median	Referral-to-death	Good (28)
Brown, 2016 [93]	USA	Very high	4b	RO	7209	U	71^‡§^	53%^§^	81%^§^	Non-malignant	SPCU	National	9*, 45^‡^	Median and mean	Length-of-stay	Good (29)
Cheraghlou, 2016 [94]	USA	Very high	4b	PO	241	1%	88^‡^	65%	90%	Mixed	Combined	Regional	15*	Median	Referral-to-death	Fair (26)
Diamond, 2016 [95]	USA	Very high	4b	RO	160	U	63^‡^	42%	62%	Malignant	Community/home	Local	44.3^‡^	Mean	Referral-to-death	Good (30)
Hamano, 2016 [96]	Japan	Very high	4b	PO	2069	2%	70^‡^	42%	U	Malignant	Combined	National	28*	Median	Survival	Fair (26)
Jarosek, 2016 [97]	USA	Very high	4b	RO	30,629	0%	U	51%	81%	Malignant	Combined	National	18*	Median	Referral-to-death	Good (31)
Jegier, 2016 [98]	USA	Very high	4b	RO	197	26%	66^‡^	37%	36%	Mixed	SPCU	Local	4.86*^†^	Median	Length-of-stay	Good (31)
Kierner, 2016 [99]	Austria	Very high	4b	RO	50	0%	62*	62%	U	Malignant	SPCU	Local	21*	Median	Length-of-stay	Fair (27)
King, 2016 [100]	USA	Very high	4b	RO	158	U	63*^†§^	55%^§^	U	Malignant	SPCU	Local	30.24*^†^	Median	Length-of-stay	Good (29)
Lowe, 2016 [101]	Canada	Very high	4b	RO	2922	0%	73^‡^	47%	U	Mixed	Combined	Regional	34*	Median	Survival	Good (29)
Masman, 2016 [102]	Netherlands	Very high	4a	PO	58	47%	75*	55%	U	Mixed	SPCU	Local	36*	Median	Length-of-stay	Good (29)
Obermeyer, 2016 [103]	USA	Very high	4b	RO	254,729	0%	78^‡§^	50%^§^	U	Malignant	Combined	National	15*	Median	Referral-to-death	Fair (24)
Odejide, 2016 [104]	USA	Very high	4b	RO	7810	0%	U	55%	93%	Malignant	Combined	National	12.055*^†^	Median	Referral-to-death	Fair (26)
Perri, 2016 [105]	Canada	Very high	4b	RO	235	4%	84^‡^	54%	U	Non-malignant	SPCU	Local	30^‡^	Mean	Length-of-stay	Fair (27)
Porteous, 2016 [106]	UK	Very high	4b	RO	298	56%	U	55%	U	Mixed	SPCU	Local	9*	Median	Length-of-stay	Good (28)
Rosenwax, 2016 [107]	Australia	Very high	4b	RO	5932	0%	76^‡§^	46%	U	Mixed	Combined	Regional	25*	Median	Referral-to-death	Good (28)
Sathornviriyapong, 2016 [108]	Thailand	High	3a	RO	317	28%	63*	49%	U	Malignant	Combined	Local	33*	Median	Survival	Good (29)
Schmalz, 2016 [109]	Germany	Very high	4b	PO	269	≥ 4%	70^‡^	42%	U	Mixed	SPCU	Local	9.7^‡^	Mean	Length-of-stay	Fair (27)
Schur, 2016 [110]	Austria	Very high	4b	RO	2414	0%	73*	52%	U	Mixed	Combined	National	9*	Median	Referral-to-death	Fair (27)
Senderovich, 2016 [111]	Canada	Very high	4b	RO	237	U	79^‡^	59%	U	Mixed	SPCU	Local	54.05^‡†^	Mean	Length-of-stay	Good (30)
Sharma, 2016 [112]	USA	Very high	4b	RO	567	0%	70^‡^	2%	94%	Malignant	Combined	Local	22.14^‡†^	Mean	Referral-to-death	Fair (27)
Stevenson, 2016 [113]	USA	Very high	4b	RO	5,519,849	19%	83^‡^	59%	89%	Mixed	Combined	National	18.91*^†^, 82.19^‡†^	Median and mean	Referral-to-death	Good (29)
United States Renal Data System, 2017 [114]	USA	Very high	4b	RO	180,491	0%	69^‡§^	54%^§^	67%^§^	Non-malignant	Combined	National	5*	Median	Referral-to-death	Fair (19)
Adsersen, 2017 [115]	Denmark	Very high	4a	RO	21,597	0%	70^‡^	50%	U	Malignant	Combined	National	29*, 64.6^‡^	Median and mean	Referral-to-death	Good (28)
Chan, 2017 [116]	UK	Very high	4b	RO	165	8%	U	U	U	Mixed	SPCU	Local	6.6^‡^	Mean	Length-of-stay	Fair (20)
Choi, 2017 [117]	South Korea	Very high	3a	RO	1829	0%	51^‡^	63%	U	Malignant	SPCU	National	26.2^‡^	Mean	Length-of-stay	Fair (25)
de la Cruz, 2017 [118]	USA	Very high	4b	RO	329	76%	56^‡^	55%	63%	Malignant	SPCU	Local	7.02^‡†^	Mean	Length-of-stay	Fair (26)
Einstein, 2017 [119]	USA	Very high	4b	RO	66	0%	64^‡^	28%	92%	Malignant	Combined	Local	93.24*^†^, 144.12^‡†^	Median and mean	Referral-to-death	Fair (26)
Forst, 2017 [120]	USA	Very high	4b	RO	7849	0%	72^‡^	49%	86%	Malignant	Combined	National	21*	Median	Referral-to-death	Good (28)
Fukui, 2017 [121]	USA	Very high	4b	RO	37,165	15%	82^‡^	60%	87%	Non-malignant	Combined	National	6.88*^†^, 17.41^‡†^	Median and mean	Referral-to-death	Fair (27)
Harris, 2017 [122]	USA	Very high	4b	RO	1972	0%	81*^§^	51%^§^	82%^§^	Mixed	Combined	National	41.04^‡†^	Mean	Referral-to-death	Fair (27)
Hoverman, 2017 [123]	USA	Very high	4b	PI	345	0%	U	U	U	Malignant	Combined	National	18.93*^†^	Median	Referral-to-death	Fair (20)
Kaufman, 2017 [124]	USA	Very high	4b	RO	1248	23%	78^‡^	54%	70%	Mixed	Combined	National	27*, 86^‡^	Median and mean	Referral-to-death	Fair (26)
Kelly, 2017 [125]	USA	Very high	4b	RO	81	0%	59*^§^	35%^§^	U	Non-malignant	Combined	Local	15*	Median	Survival	Fair (27)
Kuchinad, 2017 [126]	USA	Very high	4b	RO	39	0%	62*	36%	87%	Malignant	Combined	Local	22*	Median	Referral-to-death	Fair (25)
Lin, 2017 [127]	Taiwan	Very high	4a	RO	22,720	0%	66^‡^	40%	U	Malignant	Combined	National	9.93^‡†^	Mean	Referral-to-death	Fair (26)
Lustbader, 2017 [128]	USA	Very high	4b	RO	293	0%	86^‡^	51%	93%	Mixed	Combined	Regional	22.87*^†^, 47.07^‡†^	Median and mean	Referral-to-death	Fair (22)
Masel, 2017 [129]	Austria	Very high	4b	RO	91	25%	62*	39%	U	Malignant	SPCU	Local	16*	Median	Length-of-stay	Fair (25)
Mercadante, 2017 [130]	Italy	Very high	4b	PO	314	96%	66^‡^	42%	U	Malignant	SPCU	Local	7.01^‡†^	Mean	Length-of-stay	Good (28)
O’Leary, 2017 [17]	Ireland	Very high	4b	RO	507	0%	U	50%	U	Mixed	Combined	Regional	70^‡^	Mean	Referral-to-death	Good (29)
Otsuka, 2017 [131]	Japan	Very high	4b	RO	415	0%	72*	40%	U	Malignant	Combined	Local	54*	Median	Referral-to-death	Fair (27)
Palmer, 2017 [132]	USA	Very high	4b	RO	3704	0%	U	59%	78%	Mixed	SPCU	Local	17.5*	Median	Length-of-stay	Fair (27)
Pellizzari, 2017 [133]	Italy	Very high	4b	RO	2211	0%	U	42%	U	Malignant	Community/home	Regional	92*	Median	Referral-to-death	Good (29)
Rivet, 2017 [134]	USA	Very high	4b	RO	82	67%	60*	U	U	Non-malignant	General hospital ward	Local	1*	Median	Referral-to-death	Good (30)
Sanoff, 2017 [135]	USA	Very high	4b	RO	5056	0%	73*	31%	72%	Malignant	Combined	National	18*	Median	Referral-to-death	Fair (25)
Scaccabarozzi, 2017 [136]	Italy	Very high	4b	RO	1114	0%	76*	44%	U	Mixed	Combined	Regional	31*	Median	Referral-to-death	Fair (27)
Schuler, 2017 [137]	USA	Very high	4b	RO	7590	7%	U	51%	89%	Malignant	Combined	National	12*	Median	Referral-to-death	Good (29)
Senel, 2017 [138]	Turkey	High	3b	PO	213	44%	60^‡^	41%	U	Malignant	SPCU	Local	8.64^‡†^	Mean	Length-of-stay	Fair (27)
Shah, 2017 [139]	Australia	Very high	4b	RO	74	11%	75^‡^	19%	U	Malignant	Unspecified	Local	179.4*	Median	Survival	Good (29)
Sharp, 2017 [140]	UK	Very high	4b	RO	108	U	76^‡^	32%	U	Non-malignant	Combined	Local	124*	Median	Referral-to-death	Fair (23)
Tanuseputro, 2017 [19]	Canada	Very high	4b	RO	92,276	0%	U	51%	U	Mixed	Combined	Regional	111.1^‡^	Mean	Referral-to-death	Good (29)
Taylor, 2017 [141]	USA	Very high	4b	RO	2642	0%	U	U	77%^§^	Malignant	Combined	Regional	20*	Median	Referral-to-death	Fair (27)
Unroe, 2017 [142]	USA	Very high	4b	RO	32,605	15%	80^‡^	61%	75%	Mixed	Community/home	National	20*, 70.4^‡^	Median and mean	Referral-to-death	Good (28)
Vayne-Bossert, 2017 [143]	Australia	Very high	4b	RO	62	0%	88*^§^	U	U	Malignant	Combined	Local	103*	Median	Referral-to-death	Good (29)
Vinant, 2017 [144]	France	Very high	4b	PO	744	26%	72^‡^	48%	U	Mixed	Combined	Regional	22*	Median	Survival	Good (30)
Waite, 2017 [145]	USA	Very high	4b	RO	49	0%	78*^§^	51%^§^	U	Non-malignant	General hospital ward	Local	1.54*	Median	Referral-to-death	Fair (24)
Wang, Hsu, 2017 [146]	USA	Very high	4b	RO	71,184	0%	78^‡§^	53%^§^	87%^§^	Malignant	Combined	National	10.9^‡^	Mean	Referral-to-death	Good (29)
Wang, Knight, 2017 [147]	USA	Very high	4b	RO	394	U	U	U	U	Malignant	Combined	Regional	14.5*, 31.6^‡^	Median and mean	Referral-to-death	Good (29)
Wilson, 2017 [148]	USA	Very high	4b	RO	28	0%	75*^§^	40%^§^	U	Non-malignant	General hospital ward	Local	0.42*	Median	Referral-to-death	Good (32)
Yim, 2017 [149]	USA	Very high	4b	RO	5073	9%	82^‡^	55%	87%	Non-malignant	Combined	National	15*, 71^‡^	Median and mean	Referral-to-death	Fair (27)
Akdogan, 2018 [150]	Turkey	High	3b	RO	305	57%	80^‡^	46%	U	Mixed	SPCU	Local	31.1^‡^	Mean	Length-of-stay	Fair (26)
Allsop, 2018 [8]	UK	Very high	4b	RO	42,372	0%	U	48%	75%	Mixed	Combined	National	48*	Median	Referral-to-death	Good (30)
Assareh, 2018 [151]	Australia	Very high	4b	RO	24,468	0%	U	45%^§^	U	Mixed	General hospital ward	Regional	6*, 7.6^‡^	Median and mean	Referral-to-death	Good (30)
Cho, 2018 [152]	Singapore	Very high	4b	RO	25	0%	68^‡§^	65%^§^	U	Non-malignant	Combined	Local	8*	Median	Referral-to-death	Good (28)
Choi, 2018 [153]	South Korea	Very high	3a	RO	1829	0%	68^‡^	45%	U	Malignant	SPCU	National	26.2^‡^	Mean	Length-of-stay	Good (30)
de Oliveira Valentino, 2018 [154]	Brazil	High	3a	RO	839	0%	62^‡§^	45%^§^	U	Malignant	Combined	Local	185.54^‡^	Mean	Referral-to-death	Good (30)
Dinҫer, 2018 [155]	Turkey	High	3b	RO	85	46%	84^‡^	55%	U	Non-malignant	SPCU	Local	15*	Median	Length-of-stay	Fair (26)
Duff, 2018 [156]	USA	Very high	4b	RO	52	0%	66^‡^	2%	75%	Malignant	Unspecified	Local	21.7*	Median	Referral-to-death	Good (30)
Dunn. 2018 [157]	USA	Very high	4b	RO	96	0%	76^‡§^	3%^§^	U	Mixed	Combined	Local	24^‡^	Mean	Referral-to-death	Fair (22)
Gainza-Miranda, 2018 [158]	Spain	Very high	4a	PO	60	30%	74^‡^	20%	U	Non-malignant	Community/home	Local	252.49*	Median	Survival	Good (28)
Gidwani-Marszowski, 2018 [159]	USA	Very high	4b	RO	1,467,835	0%	U	0%	90%^§^	Mixed	Combined	National	14.17*^†^	Median	Referral-to-death	Good (30)
Gill, 2018 [160]	USA	Very high	4b	PO	244	0%	87^‡^	65%	89%	Mixed	Combined	Regional	12.5*, 40.2^‡^	Median and mean	Referral-to-death	Good (29)
Gurau, 2018 [161]	Canada	Very high	4b	RO	316	0.003%	85^‡^	52%	U	Mixed	SPCU	Local	25.62^‡†^	Mean	Length-of-stay	Fair (25)
Hattori, 2018 [162]	Japan	Very high	4b	PO	1924	0%	68*	43%	U	Malignant	SPCU	Local	38.5^‡^	Mean	Length-of-stay	Fair (26)
Hausner, 2018 [163]	Canada	Very high	4b	RO	156	0%	66*^§^	47%^§^	U	Malignant	SPCU	Local	8*	Median	Length-of-stay	Fair (25)
Hung, 2018 [164]	Taiwan	Very high	4a	RO	97,614	0%	U	38%	U	Malignant	Combined	National	28*, 62.12^‡^	Median and mean	Referral-to-death	Fair (26)
Hutchinson, 2018 [165]	USA	Very high	4b	RO	5298	0%	U	32%	98%	Malignant	Combined	National	17*	Median	Referral-to-death	Good (29)
Johnson, 2018 [166]	USA	Very high	4b	RO	101	28%	57*^§^	53%^§^	U	Non-malignant	Combined	National	42*	Median	Referral-to-death	Fair (26)
Kaufman, 2018 [167]	USA	Very high	4b	RO	43,737	U	U	56%	U	Mixed	Combined	National	26*, 70^‡^	Median and mean	Referral-to-death	Fair (24)
LeBlanc, 2018 [168]	USA	Very high	4b	RO	9230	0%	79*^§^	44%^§^	87%^§^	Malignant	Combined	National	9*	Median	Referral-to-death	Fair (27)
Ledoux, 2018 [169]	France	Very high	4b	RO	1	0%	69^‡§^	38%^§^	U	Malignant	General hospital ward	Local	1*, 1^‡^	Median and mean	Referral-to-death	Fair (27)
Lo, 2018 [170]	Canada	Very high	4b	RO	780	4%	80^‡^	52%	U	Mixed	SPCU	Local	32.41^‡†^	Mean	Length-of-stay	Fair (27)
McDermott, 2018 [171]	USA	Very high	4b	RO	8351	0.6%	77^‡§^	49%^§^	86%^§^	Malignant	Combined	National	14*	Median	Referral-to-death	Fair (27)
Mendieta, 2018 [172]	USA	Very high	4b	RO	54,105	0%	83^‡^	49%	71%	Mixed	Combined	National	7.05^‡†^	Mean	Referral-to-death	Fair (23)
Merchant, 2018 [173]	Canada	Very high	4b	RO	25,446	0%	71^‡^	40%	U	Malignant	Combined	Regional	76^§^	Median	Referral-to-death	Fair (26)
Mulville, 2018 [174]	USA	Very high	4b	RO	161	0%	74^‡^	46%	U	Malignant	Combined	Local	10*, 38^‡^	Median and mean	Referral-to-death	Poor (17)
Nazim, 2018 [175]	Canada	Very high	4b	RO	28	0%	80^‡§^	39%^§^	U	Non-malignant	General hospital ward	Local	6*	Median	Referral-to-death	Fair (26)
O'Hare, 2018 [176]	USA	Very high	4b	RO	133,962	4%	70^‡§^	47%^d^	65%^d^	Non-malignant	Combined	National	6*, 23.48^‡^	Median and mean	Referral-to-death	Fair (24)
Rozman, 2018 [177]	Brazil	High	3a	RO	2985	0%	64^‡^	45%	U	Malignant	Combined	Local	34*, 72.3^‡^	Median and mean	Referral-to-death	Fair (26)
Shih, 2018 [178]	Taiwan	Very high	4a	RO	3931	0%	72*^§^	35%^§^	U	Mixed	Combined	Local	12.69*^†^	Median	Referral-to-death	Fair(26)
Shinall Jr, 2018 [179]	USA	Very high	4b	RO	3321	50%	68^‡^	48%	79%	Mixed	SPCU	Local	3*	Median	Length-of-stay	Fair (27)
Stephens, 2018 [180]	USA	Very high	4b	RO	299	0%	56*^§^	51%^§^	U	Mixed	General hospital ward	Local	3*	Median	Referral-to-death	Good (28)
Vogl, 2018 [181]	Germany	Very high	4b	RO	784	42%	71^‡^	55%	U	Mixed	SPCU	Local	9.8^‡^	Mean	Length-of-stay	Fair (27)
Wadhwa, 2018 [182]	Canada	Very high	4b	RO	337	0%	65^‡^	48%	U	Malignant	Community/home	Local	103.41*, 206.83^‡^	Median and mean	Referral-to-death	Good (28)
Yennurajalingam, 2018 [183]	USA	Very high	4b	RO	340	0%	62*	47%	71%	Malignant	Community/home	Local	152.08*	Median	Referral-to-death	Fair (27)
Yoo, 2018 [184]	South Korea	Very high	3a	RO	277	0%	62^‡§^	39%^§^	U	Malignant	Combined	Local	45.2^‡^	Mean	Referral-to-death	Good (31)
Ziegler, 2018 [185]	UK	Very high	4b	RO	1598	0%	U	49%	U	Malignant	Combined	Local	42*	Median	Referral-to-death	Good (29)
Chai (personal commu-nication, Coyte PC, University of Toronto)	Canada	Very high	4b	RO	81	0%	U	U	U	Malignant	Community/home	Local	65*, 111.56^‡^	Median and mean	Referral-to-death	U

*UNDP HDI* United Nations Development Programme Human Development Index, *WHPCA* Worldwide Hospice Palliative Care Alliance, *RO* retrospective observational, *U* unspecified (data neither available nor possible to calculate), *SPCU* specialist palliative care unit, *USA* United States of America, *UK* United Kingdom, *PI* prospective interventional, *PO* prospective observational

*Median

^†^Weighted due to multiple subgroups with individual summaries of duration of palliative care

^‡^Mean

^§^Value for larger cohort of study participants including those not referred to palliative care

A table summarising study characteristics can be found in Additional file 2: Table S1. The total number of study participants was 11,996,479. Eighty-eight per cent of studies were observational and retrospective, and most studies were descriptive rather than analytical. The source of publications was predominantly the USA, which accounted for 85% of studies and 97% of participants. Most studies (and 99% of participants) were from very high development countries (94%) and those with the greatest level (4b) of palliative care development (83%). Patients had a weighted mean age of 81.7 years with an equal distribution of males-to-females. Ethnicity was only reported in 38% of studies, stating 88% of study participants as white Caucasian. Most studies reported patients with malignant (50%) or non-malignant disease (12%). However, studies that reported a combined case-mix (35%) covered 91% of total participants. Similarly, half of the studies reported on specific types of palliative care services (31% SPCU, 11% community/home and 7% hospital), with studies reporting in combined settings (50%) accounting for 95% of all participants.

Of all included articles, 46 (27%) were length-of-stay studies. The proportion of patients alive at the end of each study is outlined in Table 1. In 28 of the length-of-stay studies (60.9%) fewer than 10% of patients were alive, with 22 (47.8%) having no patients alive at the end of study.

Study quality was rated as good in 73 (43%) studies, fair in 90 (53%) and poor in 5 (3%). Studies rated as good accounted for 64% of total participants although studies variably summarised the duration of palliative care with inconsistent measures of spread. A table of individual study quality appraisals can be found in Additional file 2: Table S3.

The weighted median duration of palliative care until death was 18.9 days (IQR 0.09, Table *2*). Three studies had more than one million participants each [48, 113, 159]. The median duration of palliative care excluding these studies (total 16.7% participants) was 19.2 days (IQR 15). The weighted median duration in days until death per country, by service type, disease type, WHPCA level of palliative care development, and UNDP Human Development Index is reported in Table 2.

**Table 2:** Duration of palliative care in days prior to death, by country and other characteristics

		Weighted median duration of palliative care in days (IQR)	*p* value
All studies		18.91 (0.09)	
Country	Service type		
Australia		6.00 (8.81)	
	Specialist palliative care	14.81 (0.00)	
	Community/home	-	
	General hospital ward	6.00 (0.00)	
	Combined	25.00 (0.00)	
Austria		9.00 (0.00)	
	Specialist palliative care	16.00 (5.00)	
	Community/home	-	
	General hospital ward	-	
	Combined	9.00 (0.00)	
Belgium		17.95 (*^†^)	
	Specialist palliative care	-	
	Community/home	-	
	General hospital ward	-	
	Combined	17.95 (0.00)	
Brazil		34.00 (0)	
	Specialist palliative care	-	
	Community/home	115.02 (0.00)	
	General hospital ward	-	
	Combined	34.00 (0.00)	
Canada		68.88 (0.00)	
	Specialist palliative care	25.60 (3.73)	
	Community/home	47.92 (0.00)	
	General hospital ward	6.00 (0.00)	
	Combined	68.88 (0.00)	
China		19.00 (*^†^)	
	Specialist palliative care	19.00 (0.00)	
	Community/home	-	
	General hospital ward	-	
	Combined	-	
Denmark		29.00 (*^†^)	
	Specialist palliative care	-	
	Community/home	-	
	General hospital ward	-	
	Combined	29.99 (0.00)	
Egypt		66.00 (*^†^)	
	Specialist palliative care	66.00 (0.00)	
	Community/home	-	
	General hospital ward	-	
	Combined	-	
Finland		25.37 (*^†^)	
	Specialist palliative care	25.37 (0.00)	
	Community/home	-	
	General hospital ward	-	
	Combined	-	
France		22.00 (0.00)	
	Specialist palliative care	-	
	Community/home	-	
	General hospital ward	-	
	Combined	22.00 (0.00)	
Germany		13.16 (0)	
	Specialist palliative care	13.16 (0.00)	
	Community/home	-	
	General hospital ward	-	
	Combined	-	
Ireland		46.27 (0.00)	
	Specialist palliative care	-	
	Community/home	-	
	General hospital ward	10.30 (0.00)	
	Combined	46.27 (0.00)	
Italy		21.00 (10.00)	
	Specialist palliative care	11.62 (6.26)	
	Community/home	92.00 (0.00)	
	General hospital ward	-	
	Combined	21.00 (0.00)	
Japan		28.95 (0.95)	
	Specialist palliative care	28.95 (0.00)	
	Community/home	35.00 (0.00)	
	General hospital ward	-	
	Combined	28.00 (0.00)	
Netherlands		36.00 (37^†^)	
	Specialist palliative care	36.00 (0.00)	
	Community/home	-	
	General hospital ward	-	
	Combined	-	
Saudi Arabia		16.63 (*^†^)	
	Specialist palliative care	-	
	Community/home	-	
	General hospital ward	-	
	Combined	16.63 (0.00)	
Singapore		8.00 (19^†^)	
	Specialist palliative care	-	
	Community/home	-	
	General hospital ward	-	
	Combined	8.00 (0.00)	
South Korea		22.18 (0.00)	
	Specialist palliative care	22.18 (0.00)	
	Community/home	-	
	General hospital ward	-	
	Combined	32.63 (0.00)	
Spain		43.52 (0.00)	
	Specialist palliative care	-	
	Community/home	43.52 (0.00)	
	General hospital ward	-	
	Combined	-	
Taiwan		28.00 (0.00)	
	Specialist palliative care	13.50 (0.00)	
	Community/home	-	
	General hospital ward	33.71 (0.00)	
	Combined	28.00 (0.00)	
Thailand		33.00 (*^†^)	
	Specialist palliative care	-	
	Community/home	-	
	General hospital ward	-	
	Combined	33.00 (0.00)	
Turkey		24.88 (12.35)	
	Specialist palliative care	24.88 (12.35)	
	Community/home	-	
	General hospital ward	-	
	Combined	-	
UK		48.00 (0.00)	
	Specialist palliative care	9.00 (2.40)	
	Community/home	41.41 (294.59)	
	General hospital ward	-	
	Combined	48.00 (0.00)	
Multicentre – USA & Brazil		6.00 (5^†^)	
	Specialist palliative care	6.00 (0.00)	
	Community/home	-	
	General hospital ward	-	
	Combined	-	
USA		18.91 (0.09)	
	Specialist palliative care	19.20 (17.72)	
	Community/home	20.00 (0.00)	
	General hospital ward	3.00 (12.01)	
	Combined	18.91 (0.09)	
USA vs non-USA studies	USA	18.91 (0.09)	*p* < 0.001
	All non-USA studies	29.00 (40.88)	
UNDP Human Development Index (2015)	Very high	18.91 (0.09)	
	High	34.00 (0)	
	Medium	66.00 (*^†^)	
	No data	6.00 (5^†^)	
	Very high	18.91 (0.09)	*p* < 0.001
	< Very high	34.00 (1.00)	
WHPCA categorisation of palliative care development (2011)	4b	18.91 (0.09)	
	4a	28.00 (0)	
	3b	24.88 (12.35)	
	3a	22.18 (11.82)	
	No data	6.00 (5^†^)	
	4b	18.91 (0.09)	*p* < 0.001
	< 4b	28.00 (0)	
Type of disease	Malignant	15.00 (7.18)	*p* < 0.001
	Non-malignant	6.00 (1.00)	
	Mixed	18.91 (0.09)	
	Unspecified	14.81 (0)	
Type of palliative care service	Specialist palliative care unit	19.20 (17.72)	*p* < 0.001
	Community/home	20.00 (0)	
	General hospital ward	6.00 (0)	
	Combined	18.91 (0.09)	
	Unspecified	21.70 (167.40)	

Median data weighted against the size of the study population of included studies

*IQR* interquartile range, *UK* United Kingdom, *USA* United States of America

*Data neither available nor possible to calculate

^†^IQR from single study sample

Analyses of the influence of study characteristics on the overall weighted median duration of palliative care until death are outlined in Additional file 2: Table S4. Studies rated as poor and fair did not significantly adjust the duration of palliative care for the whole dataset, but they significantly reduced the duration of palliative care when looking at non-USA studies alone. Studies tended to report longer mean than median durations where both were used, reflecting positively skewed data. Studies in which mean durations were converted to medians did not affect the outcome for the whole dataset, but did significantly increase the duration of palliative care for non-USA studies. Even excluding studies with more than one million participants did not greatly alter the duration of palliative care (i.e. 19.2 days).

**Fig. 2:**
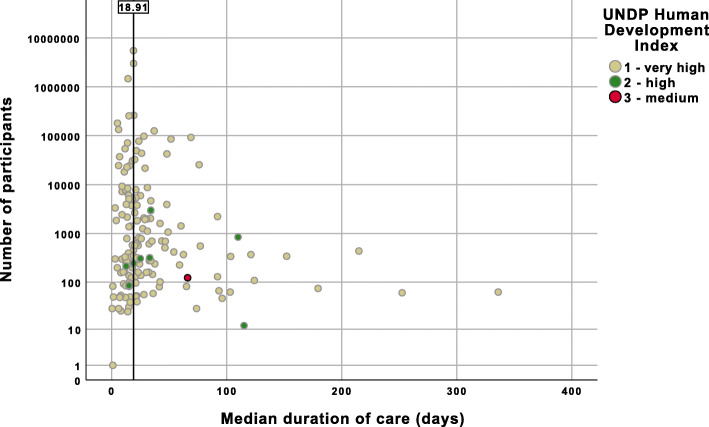
Scatter plot showing individual studies according to sample size, median duration of palliative care and UNDP Human Development Index

**Fig. 3:**
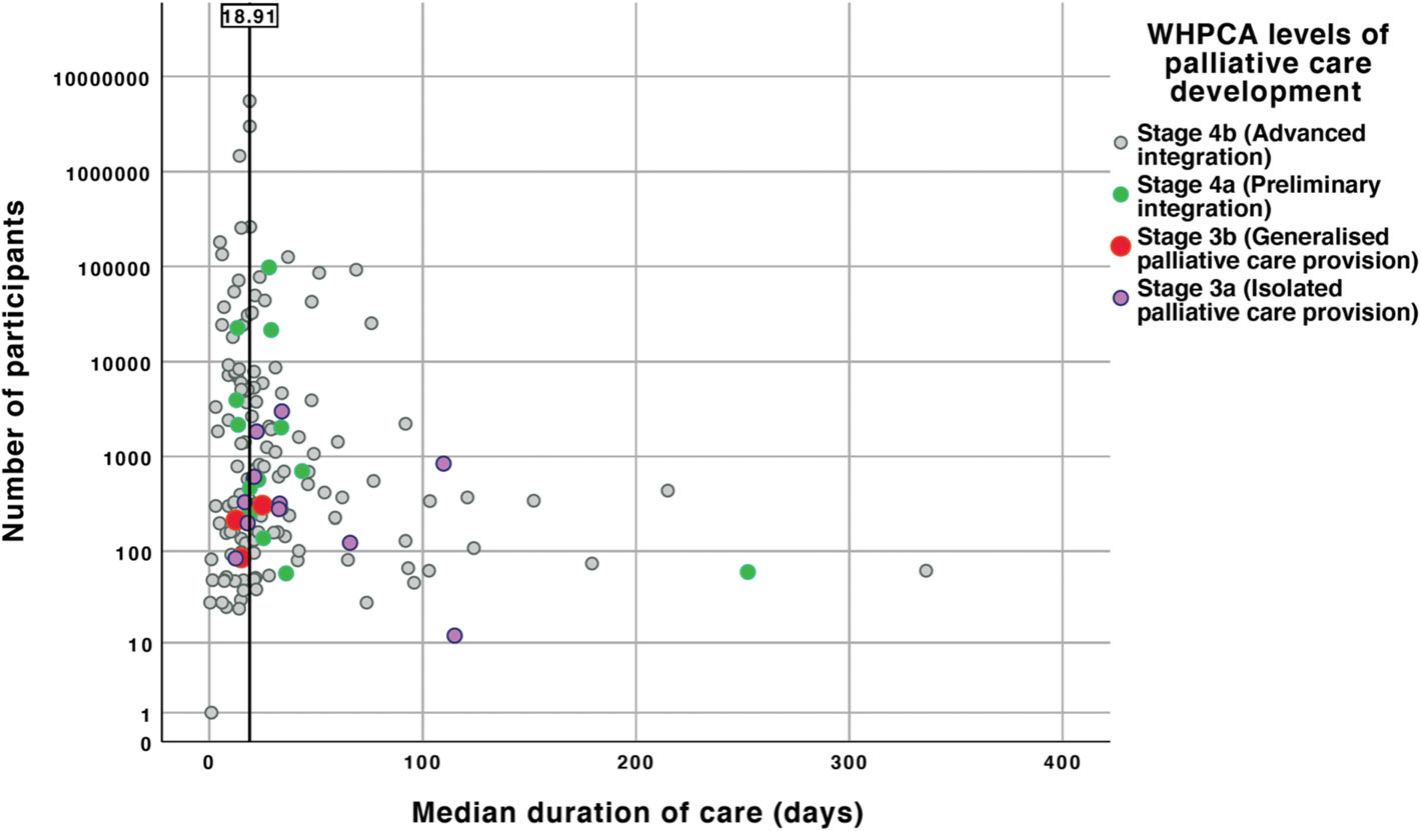
Scatter plot showing individual studies according to sample size, median duration of palliative care and WHPCA categorisation of palliative care development

The spread of median duration of palliative care values according to sample size is demonstrated in Figs. 2 and 3.

Figure 2 outlines the difference in median days duration of palliative care prior to death according to country level of human development and palliative care development. Studies from countries with a very high level of human development had a shorter duration of palliative care than less developed countries (18.9 vs. 34.0 days, *p* < 0.001). Similarly, as shown in Fig. 3, studies from countries with the greatest level of palliative care development had a shorter duration of palliative care than countries with lower levels (18.9 vs. 28.0 days, *p* < 0.001). Not all studies reported duration of palliative care for patients with both malignant and non-malignant disease, and across a combination of palliative care settings. Consequently, we conducted sub-analyses using data on the type of disease available in 105 of the included studies (i.e. 62.1%) and the type of palliative care setting using data from 82 studies (i.e. 48.5%). The median duration of palliative care was nine days longer for studies reporting on patients with malignant disease compared with non-malignant disease (15.0 vs. 6.0, *p* < 0.001). Studies conducted in specialist palliative care units and community/home settings reported a similar duration of palliative care, both longer than in general hospital ward settings (19.2 vs. 20.0 vs. 6.0 days, respectively, *p* < 0.001). A further sub-analysis, comparing data from the USA and non-USA countries, was performed given the preponderance of data from the former. The median duration of palliative care in studies from the USA was ten fewer days than in non-USA studies (18.9 vs. 29.0, *p* < 0.001).

The median duration of palliative care was unaffected by studies reporting local or regional data, reporting duration of palliative care as a mean solely, reporting length-of-stay, studies with < 100 participants and studies rated as fair or poor quality. It was, however, reduced to 14.71 days (IQR 0.83; MAD 8.23) after excluding studies with > 5% participants alive at the end of the study period. Sensitivity analysis showed that studies rated as poor and fair did not significantly adjust the duration of palliative care for the whole dataset, but they significantly reduced the duration of palliative care when looking at non-USA studies alone.

Given differences between studies from and outside of the USA, we hypothesised that non-USA studies may have different factors influencing the duration of palliative care. We conducted additional analyses excluding USA data (Additional file 2: Tables S4 and S5). Studies from non-USA countries with a very high level of human development still had a shorter duration of palliative care than less developed countries (29.0 vs. 34.0 days, *p* < 0.001). However, studies from non-USA countries with the greatest level of palliative care development had a longer duration of palliative care than countries with lower levels of palliative care development (68.9 vs. 28.0 days, *p* < 0.001). Studies involving patients with malignant disease reported a longer duration of palliative care than those with non-malignant disease; however, the difference was smaller (28.0 vs. 24.3 days, *p* < 0.001). Studies conducted in community or home settings had a longer duration of palliative care than those conducted in specialist palliative care units and general hospital ward settings (47.9 vs. 14.8 vs. 6.0 days, respectively, *p* < 0.001).

The sensitivity analyses showed the median duration of palliative care for non-USA studies was unaffected by length-of-stay, studies with < 100 participants, and studies with > 5% of cohorts alive at the end of the study periods. However, it was reduced to 28.0 days after excluding local and regional studies (IQR 1.00; MAD 8.00) and studies reporting duration of palliative care as a mean solely (IQR 20.00; MAD 14.00) and was increased to 48.0 days (IQR 39.88; MAD 25.41) after excluding poor/fair quality studies.

## Discussion

In this systematic review, 169 studies were included, involving 11,996,479 patients. 43% of studies were of good quality and studies variably summarised duration of palliative care with inconsistent measures of spread. Internationally, half of all patients accessing palliative care services are referred less than 19 days before death, although we found very large diversity in the median duration of palliative care in days prior to death across the countries in this review, from 6 days in Australia to 69 days in Canada. The median number of days of palliative care prior to death for all US studies was 19 days, and for all non-US studies, it was 29 days. Cancer patients have a longer duration of palliative care as compared with those with non-malignant disease. We found palliative care duration is comparable for patients referred to specialist inpatient units and community settings, but significantly longer than for patients in a general hospital ward. At a country level, human development index level and the extent of palliative care development had an unexpected negative effect on the duration of palliative care.

This large systematic review and meta-analysis of international data found the duration of palliative care before death for patients with life-limiting illness is much shorter (i.e. a median of 19 days) than is supported by research evidence and widely advocated in health care policy. Davis et al.’s systematic review of randomised trials of early integration of outpatient and home palliative care concluded that care must be provided for at least 3–4 months before death to reach maximal benefit [5]. Although we appreciate duration and content of palliative care should be guided by individual patient needs without a one-size-fits-all approach, we are concerned that this reflects a gap between the current practice of palliative care in the terminal phase of life and the timely initiation of palliative care, which impacts on the benefit of palliative care for patients and health care services. This work extends previous efforts by the team to understand the duration of hospice-based specialist palliative care in the UK [8]. This review augments the focus to include data across multiple care settings, including hospital, home and the community, alongside novel comparisons of the duration of palliative care across countries internationally.

Variation in the duration of palliative care before death across countries reflected a range from a median of 6 days (Australia) to 69 days (Canada). Whilst this reflects only published data there is stark variation, with duration of palliative care encompassing only a few days prior to death for some countries. Data from countries may, to some extent, reflect the country-specific provision of palliative care. For example, data from the USA reflected patients receiving ten fewer days in palliative care than those in non-USA countries. This may be explained by USA models of care that restrict hospice care to patients with prognoses less than six months and require patients to stop active treatments that may still be beneficial [186]. Given high levels of palliative care development and human development of included non-USA studies, it is likely that these countries are able to offer similar life-prolonging and supportive healthcare interventions as the USA [187].

Longer duration of palliative care for patients with malignant disease compared with those with non-malignant disease, as found in this study, is consistent with a UK report that found patients with cancer were predominantly referred to palliative care services despite only accounting for 29% of deaths in 2012–2013 [188]. Allsop et al. found cancer patients had a significantly longer median duration between referral to UK hospices and death compared with non-cancer patients (53 days vs. 27 days, *p* < 0.0001) [8]. This occurs despite evidence that palliative care needs and symptom burden are comparable between cancer and non-cancer groups [189, 190]. Although evidence in support of palliative care interventions is predominantly from studies involving patients with cancer, this is emerging for non-cancer groups [4]. Siouta et al. found that guidelines and pathways supporting the integration of palliative care in major non-malignant disease are increasingly involving earlier palliative care integration but lack information on referral criteria [191]. Other barriers to accessing palliative care in this group must be understood in order to improve integration of care.

We found palliative care duration is comparable for patients referred to specialist inpatient units and community settings, but that this was significantly longer than for patients referred as general hospital inpatients. This is consistent with studies comparing the duration of palliative care between outpatient or home palliative care and general hospital settings, and probably reflects greater referrals of patients in the last days of life in the latter setting [10, 55, 64]. Hui et al. found that patients referred to outpatient palliative care had improved end of life care more than those who received inpatient palliative care from mobile teams [55]. It may be appropriate to concentrate efforts to increase the duration of palliative care in outpatient settings, prior to a longer-term goal of increasing duration of palliative care in all settings. Non-USA patients already spend fewer days in specialist palliative care units and more days in community palliative care, which may reflect patient preference, or reduced capacity of and access to inpatient settings [192].

We found a negative correlation between duration of palliative care and country level of human development. For the limited studies that were not categorised as ‘very high’ according to the United Nations Human Development Index, all but one reported data on malignant conditions. The negative correlation may, therefore, partly reflect the longer duration of palliative care for patients with malignant disease found across all studies. However, the extent to which firm conclusions can be drawn regarding the duration of palliative care and country level of human development is limited. Firstly, the predominance of malignant disease does not reflect the current multitude of diseases and symptoms that characterise health conditions requiring palliative care in the context of low and middle-income countries [6]. Secondly, no studies were included from countries classified as ‘low’ using the United Nations Human Development Index. This review highlights the wider need to support increase in research capacity in the context of low and middle-income countries (LMICs) to better understand the provision of palliative care [6]. There remains a disparity of the reporting of palliative care research in LMICs which needs to be prioritised [193]. These are the countries in which the greatest proportional rise in serious health-related suffering is projected to occur [7]. Alongside efforts to, for example, utilise routinely collected datasets to determine the temporal nature of initiating and subsequent duration of palliative care in LMICs [194], efforts to better understand optimal timing and provision of palliative care in these settings is required. It is not appropriate to extrapolate the existing evidence for early referrals, largely from high-income settings, to countries and settings in which palliative care is critically absent and largely a poverty-reduction intervention to lessen significant costs that can be absorbed by the individual, family and local community arising from incurable illnesses [195].

The main strength of this systematic review is the inclusion of a large number of studies with over 11 million participants, giving significant power to our findings. Duration of palliative care was difficult to define due to the range of different palliative care settings and terminology used to describe this outcome measure. We used a complex search strategy including supplementary searching to identify studies that used inconsistent terminology for palliative care and duration of palliative care. It was not possible to use a statistical method to assess heterogeneity or publication bias. However, we conducted sensitivity analyses in order to further interrogate the data and explain any heterogeneity within the data. Limitations included the definition of palliative care services and the use of length-of-stay in our inclusion criteria. Individual studies were unclear on the level of training and experience of palliative care practitioners and services. Therefore, we chose to assume that services self-defining as specialist palliative care were such but may have included some studies from services with less specialist experience. We used length-of-stay in an inpatient specialist palliative care setting as a proxy for the duration of palliative care as many patients are first referred to these settings and die during first admissions. Across half of all length-of-stay studies included in this review, the entire study population had died at the end of the study period. However, we acknowledge that it is increasingly common for patients to have short inpatient admissions for symptom control with eventual discharge. In the UK, 32% of patients admitted to inpatient hospices are discharged [196]. As such this may not fully reflect the breadth of input from palliative care services and patients admitted to inpatient hospices may have had earlier contact with community or hospital palliative care services. Consequently, the use of length-of-stay could underestimate the duration of palliative care. However, our sensitivity analyses showed that studies reporting length-of-stay did not significantly alter the overall duration of palliative care. Studies reporting > 5% survival at the end of the study period significantly increased the duration of palliative care for the whole dataset, suggesting that our main finding may be an overestimation.

## Conclusions

This review suggests that duration of palliative care before death for patients with life-limiting illness is much shorter than is supported by research evidence and widely advocated in health care policy. Our study also highlights wide variation at the level of country, across disease types and settings to which patients are referred. This review draws attention to the increasing extent to which palliative care research is capturing the duration and interaction provided to patients and their families. However, to better understand the timing of palliative care provision internationally, we welcome more consistent terminology and methodology, and routine assessment of duration of palliative care from all countries, to allow benchmarking, service evaluation and quality improvement. This could lead to a greater understanding of the duration of palliative care and associated factors. However, we acknowledge that further research is required across all countries to understand the mechanisms influencing differences in the duration of palliative care received, across the levels of patients, caregivers, health professionals, policymakers and the public, and the settings in which care is provided. In particular, there is a need for greater reporting in less developed settings where there is a dearth of related literature and likely to be the greatest need in future [7]. Reducing barriers to accessing palliative care and promoting earlier integration alongside active treatment would maximise benefits to patients before they die and reduce costs to the wider healthcare service.

## Supplementary Information

**Additional file 1:** **Fig S1.** Example of search strategy as used in MEDLINE. **Fig S2.** Linear regression model used to compare mean and median values.

**Additional file 2:** **Table S1.** PRISMA checklist. **Table S2.** Summary of characteristics of studies. **Table S3.** Individual study quality appraisal using Hawker’s criteria. **Table S4.** Summary of characteristics of studies (excluding USA data). **Table S5.** Duration of care with sub-analyses (excluding USA data).

## Data Availability

All data generated or analysed during this study are included in this published article and its supplementary information files.

## Abbreviations

IQR: Interquartile range
LMICs: Low- and middle-income countries
MAD: Median absolute deviation
RCTs: Randomised controlled trials
UK: United Kingdom
USA: United States of America
