# Supplementary material for: Duration of palliative care before death in international routine practice: a systematic review and meta-analysis
Source: BMC Med. 2020 Nov 26;18:368. doi: 10.1186/s12916-020-01829-x (PMC7690105; doi:10.1186/s12916-020-01829-x)
Supplement: Supplementary file 1 — Additional file 1: Fig S1. Example of search strategy as used in MEDLINE. Fig S2. Linear regression model used to compare mean and median values. [file 12916_2020_1829_MOESM1_ESM.docx]

**Fig S1. Example of search strategy as used in MEDLINE.**

**
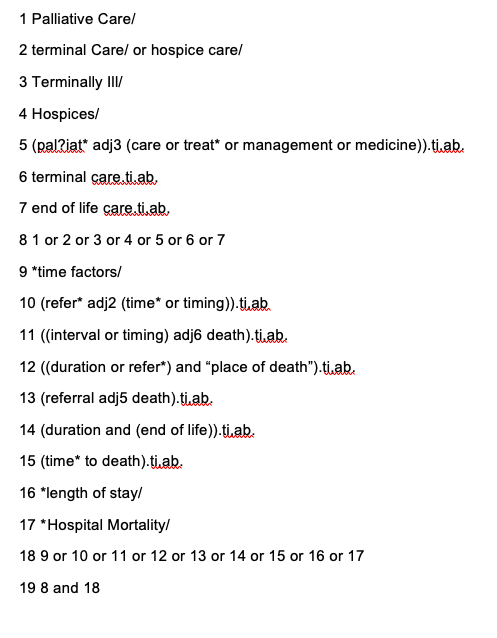
**

**Fig S2. Linear regression model used to compare mean and median values.**


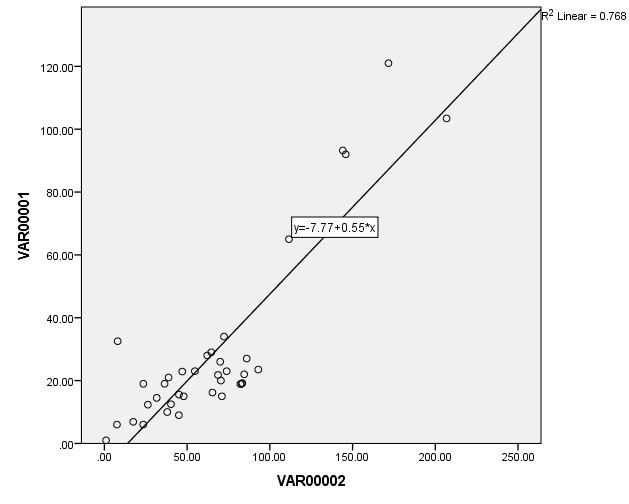


**Median**

**Mean**
