# Supplementary material for: Duration of palliative care before death in international routine practice: a systematic review and meta-analysis
Source: BMC Med. 2020 Nov 26;18:368. doi: 10.1186/s12916-020-01829-x (PMC7690105; doi:10.1186/s12916-020-01829-x)
Supplement: Supplementary file 2 — Additional file 2: Table S1. PRISMA checklist. Table S2. Summary of characteristics of studies. Table S3. Individual study quality appraisal using Hawker’s criteria. Table S4. Summary of characteristics of studies (excluding USA data). Table S5. Duration of care with sub-analyses (excluding USA data). [file 12916_2020_1829_MOESM2_ESM.docx]

**Table S1: PRISMA checklist.**

| **Section/topic** | **#** | **Checklist item** | **Reported on page #** |
| --- | --- | --- | --- |
| **TITLE** | | |  |
| Title | 1 | Identify the report as a systematic review, meta-analysis, or both. | 1 |
| **ABSTRACT** | | |  |
| Structured summary | 2 | Provide a structured summary including, as applicable: background; objectives; data sources; study eligibility criteria, participants, and interventions; study appraisal and synthesis methods; results; limitations; conclusions and implications of key findings; systematic review registration number. | 2 |
| **INTRODUCTION** | | |  |
| Rationale | 3 | Describe the rationale for the review in the context of what is already known. | 4 |
| Objectives | 4 | Provide an explicit statement of questions being addressed with reference to participants, interventions, comparisons, outcomes, and study design (PICOS). | 4 |
| **METHODS** | | |  |
| Protocol and registration | 5 | Indicate if a review protocol exists, if and where it can be accessed (e.g., Web address), and, if available, provide registration information including registration number. | 5 |
| Eligibility criteria | 6 | Specify study characteristics (e.g., PICOS, length of follow-up) and report characteristics (e.g., years considered, language, publication status) used as criteria for eligibility, giving rationale. | 5 |
| Information sources | 7 | Describe all information sources (e.g., databases with dates of coverage, contact with study authors to identify additional studies) in the search and date last searched. | 5 |
| Search | 8 | Present full electronic search strategy for at least one database, including any limits used, such that it could be repeated. | 5 (Supplemental material Figure 1) |
| Section/topic | # | Checklist item | Reported on page # |
| Study selection | 9 | State the process for selecting studies (i.e., screening, eligibility, included in systematic review, and, if applicable, included in the meta-analysis). | 5 |
| Data collection process | 10 | Describe method of data extraction from reports (e.g., piloted forms, independently, in duplicate) and any processes for obtaining and confirming data from investigators. | 5 |
| Data items | 11 | List and define all variables for which data were sought (e.g., PICOS, funding sources) and any assumptions and simplifications made. | 6 |
| Risk of bias in individual studies | 12 | Describe methods used for assessing risk of bias of individual studies (including specification of whether this was done at the study or outcome level), and how this information is to be used in any data synthesis. | 6 |
| Summary measures | 13 | State the principal summary measures (e.g., risk ratio, difference in means). | 6 |
| Synthesis of results | 14 | Describe the methods of handling data and combining results of studies, if done, including measures of consistency (e.g., I^2^) for each meta-analysis. | 6 |
| Risk of bias across studies | 15 | Specify any assessment of risk of bias that may affect the cumulative evidence (e.g., publication bias, selective reporting within studies). | 6 |
| Additional analyses | 16 | Describe methods of additional analyses (e.g., sensitivity or subgroup analyses, meta-regression), if done, indicating which were pre-specified. | 6 |
| **RESULTS** | | |  |
| Study selection | 17 | Give numbers of studies screened, assessed for eligibility, and included in the review, with reasons for exclusions at each stage, ideally with a flow diagram. | 7 (Figure 1) |
| Study characteristics | 18 | For each study, present characteristics for which data were extracted (e.g., study size, PICOS, follow-up period) and provide the citations. | 8-13 (Table 1) |
| Risk of bias within studies | 19 | Present data on risk of bias of each study and, if available, any outcome level assessment (see item 12). | 14 (Supplemental material Table 2) |
| Results of individual studies | 20 | For all outcomes considered (benefits or harms), present, for each study: (a) simple summary data for each intervention group (b) effect estimates and confidence intervals, ideally with a forest plot. | Effect estimates are not applicable for this type of meta-analysis, individual study results shown in Table 1 (p.8-13) |
| Synthesis of results | 21 | Present results of each meta-analysis done, including confidence intervals and measures of consistency. | Summarised as median duration of care on p.14, also see Table 2 (p.15) and Figure 2 (p.16) |
| Risk of bias across studies | 22 | Present results of any assessment of risk of bias across studies (see Item 15). | 14 (Supplemental material Table 2) |
| Additional analysis | 23 | Give results of additional analyses, if done (e.g., sensitivity or subgroup analyses, meta-regression [see Item 16]). | 17 |
| **DISCUSSION** | | |  |
| Summary of evidence | 24 | Summarize the main findings including the strength of evidence for each main outcome; consider their relevance to key groups (e.g., healthcare providers, users, and policy makers). | 18 |
| Limitations | 25 | Discuss limitations at study and outcome level (e.g., risk of bias), and at review-level (e.g., incomplete retrieval of identified research, reporting bias). | 19-20 |
| Conclusions | 26 | Provide a general interpretation of the results in the context of other evidence, and implications for future research. | 20 |
| **FUNDING** | | |  |
| Funding | 27 | Describe sources of funding for the systematic review and other support (e.g., supply of data); role of funders for the systematic review. | 21 |

**Table S2: Summary of studies characteristics.**

|  | | **Number of studies**  **(%)** | **Number of patients (%)** |
| --- | --- | --- | --- |
| **All studies** | | 169 (100%) | 11,996,479 (100%) |
| **Country of origin** | Australia | 8 (4·7%) | 37,404 (0·3%) |
|  | Austria | 3 (1·8%) | 2,555 (0·0%) |
|  | Belgium | 1 (0·6%) | 577 (0·0%) |
|  | Brazil | 3 (1·8%) | 3,836 (0·0%) |
|  | Canada | 16 (9·5%) | 127,275 (1·1%) |
|  | China | 1 (0·6%) | 244 (0·0%) |
|  | Denmark | 1 (0·6%) | 21,597 (0·2%) |
|  | Egypt | 1 (0·6%) | 123 (0·0%) |
|  | Finland | 1 (0·6%) | 138 (0·0%) |
|  | France | 2 (1·2%) | 745 (0·0%) |
|  | Germany | 3 (1·8%) | 1,278 (0·0%) |
|  | Ireland | 3 (1·8%) | 647 (0·0%) |
|  | Italy | 5 (3·0%) | 9,195 (0·1%) |
|  | Japan | 4 (2·4%) | 5,101 (0·0%) |
|  | Netherlands | 1 (0·6%) | 58 (0·0%) |
|  | Saudi Arabia | 1 (0·6%) | 328 (0·0%) |
|  | Singapore | 1 (0·6%) | 25 (0·0%) |
|  | South Korea | 6 (3·6%) | 4,826 (0·0%) |
|  | Spain | 2 (1·2%) | 758 (0·0%) |
|  | Taiwan | 7 (4·1%) | 129,467 (1·1%) |
|  | Thailand | 1 (0·6%) | 317 (0·0%) |
|  | Turkey | 3 (1·8%) | 603 (0·0%) |
|  | UK | 9 (5·3%) | 50,021 (0·4%) |
|  | Multicentre – USA & Brazil | 1 (0·6%) | 357 (0·0%) |
|  | USA | 85 (50·3%) | 11,599,004 (96·7%) |
| **UNDP Human Development Index (2015)** | Very high | 159 (94·1%) | 11,990,999 (99·9%) |
|  | High | 8 (4·7%) | 5,000 (0·0%) |
|  | Medium | 1 (0·6%) | 123 (0·0%) |
|  | Low | 0 (0%) | 0 (0%) |
|  | Other | 0 (0%) | 0 (0%) |
|  | No data | 1 (0·6%) | 357 (0·0%) |
| **WHPCA categorisation of palliative care development (2011)** | 4b | 140 (82·8%) | 11,833,827 (98·6%) |
|  | 4a | 13 (7·7%) | 152,262 (1·3%) |
|  | 3b | 3 (1·8%) | 603 (0·0%) |
|  | 3a | 12 (7·1%) | 9,430 (0·1%) |
|  | 2 | 0 (0%) | 0 (0%) |
|  | 1 | 0 (0%) | 0 (0%) |
|  | No data | 1 (0·6%) | 357 (0·0%) |
| **Type of disease** | Malignant | 84 (49·7%) | 665,449 (5·5%) |
|  | Non-malignant | 21 (12·4%) | 365,196 (3·0%) |
|  | Mixed | 59 (34·9%) | 10,959,028 (91·4%) |
|  | Unspecified | 5 (3·0%) | 6,806 (0·1%) |
| **Type of palliative care service** | Specialist palliative care unit | 52 (30·8%) | 584,688 (4·9%) |
|  | Community/home | 18 (10·7%) | 43,279 (0·4%) |
|  | General hospital ward | 12 (7·1%) | 27,355 (0·2%) |
|  | Combined | 84 (49·7%) | 11,340,983 (94·5%) |
|  | Unspecified | 3 (1·8%) | 174 (0·0%) |
| **Level of analysis** | Local | 85 (50·3%) | 39,821 (0·3%) |
|  | Regional | 30 (17·8%) | 240,162 (2·0%) |
|  | National | 53 (31·4%) | 11,716,139 (97·7%) |
|  | International | 1 (0·6%) | 357 (0·0%) |
| **Statistical summary used to report duration of care** | Solely mean | 43 (25·4%) | 530,696 (4·4%) |
|  | Solely median | 89 (52·7%) | 2,224,236 (18·5%) |
|  | Median and mean | 37 (21·9%) | 9,241,547 (77·0%) |
| **Terminology used to report duration of care** | Referral-to-death | 101 (59·8%) | 11,466,292 (95·6%) |
|  | Survival | 16 (9·5%) | 14,281 (0·1%) |
|  | Length-of-stay | 52 (30·8%) | 515,906 (4·3%) |
| **No. study participants** | <100 | 36 (21·3%) | 2,004 (0·0%) |
|  | ≥100 | 133 (78·7%) | 11,994,475 (99·9%) |
| **Participants alive at the end of the study period** | ≤5% | 110 (65·1%) | 2,914,522 (24·3%) |
|  | >5% | 59 (34·9%) | 9,081,957 (75·7%) |
| **Hawker’s criteria rating** | Poor | 5 (3·0%) | 4,046 (0·0%) |
|  | Fair | 90 (53·3%) | 4,334,426 (36·1%) |
|  | Good | 73 (43·2%) | 7,657,926 (63·8%) |
|  | Missing* | 1 (0·6%) | 81 (0·0%) |

**Table S3: Individual study quality appraisals using Hawker’s criteria.**

|  | **Abstract and title rating** | **Introduction and aims rating** | **Method and data rating** | **Sampling rating** | **Data analysis rating** | **Ethics and bias rating** | **Results rating** | **Transferability/generalizability rating** | **Implications and usefulness rating** | **Overall rating (total score)** |
| --- | --- | --- | --- | --- | --- | --- | --- | --- | --- | --- |
| Aeckerle, 2013 (22) | Good | Poor | Fair | Poor | Good | Very poor | Good | Poor | Good | Fair (26) |
| Alsirafy, 2013 (23) | Good | Good | Poor | Very poor | Poor | Poor | Fair | Poor | Fair | Fair (23) |
| Bakitas, 2013 (24) | Fair | Poor | Fair | Poor | Good | Poor | Good | Poor | Good | Fair (26) |
| Beernaert, 2013 (25) | Good | Fair | Fair | Poor | Good | Poor | Good | Poor | Fair | Fair (27) |
| Cheung, 2013 (26) | Fair | Fair | Fair | Poor | Good | Good | Good | Poor | Good | Good (29) |
| Corbett, 2013 (27) | Poor | Fair | Poor | Poor | Good | Very poor | Good | Poor | Poor | Fair (22) |
| D’Angelo, 2013 (28) | Poor | Fair | Fair | Poor | Good | Good | Good | Poor | Fair | Fair (27) |
| Dong, 2013 (29) | Poor | Fair | Fair | Poor | Good | Good | Good | Poor | Good | Good (28) |
| Eti, 2013 (30) | Poor | Poor | Fair | Poor | Good | Poor | Good | Poor | Fair | Fair (24) |
| Gerber, 2013 (31) | Poor | Poor | Very poor | Poor | Poor | Very poor | Very poor | Poor | Poor | Poor (15) |
| Harris, 2013 (32) | Poor | Fair | Poor | Poor | Good | Poor | Good | Poor | Fair | Fair (24) |
| Hussain, 2013 (33) | Good | Good | Good | Poor | Good | Good | Good | Poor | Good | Good (32) |
| Kelley, 2013 (34) | Poor | Good | Good | Poor | Good | Very poor | Good | Poor | Fair | Fair (26) |
| Mack, 2013 (35) | Good | Fair | Good | Poor | Good | Poor | Good | Poor | Fair | Good (28) |
| Meng, 2013 (36) | Good | Good | Fair | Poor | Good | Poor | Good | Poor | Good | Good (29) |
| Mercadante, 2013 (37) | Poor | Good | Poor | Poor | Good | Fair | Good | Poor | Fair | Fair (26) |
| Nabal, 2013 (38) | Poor | Good | Poor | Fair | Good | Poor | Good | Good | Fair | Good (28) |
| Nevadunsky, 2013 (39) | Good | Good | Poor | Poor | Good | Very poor | Good | Poor | Fair | Fair (26) |
| Pattenden, 2013 (40) | Good | Good | Fair | Poor | Good | Poor | Good | Poor | Good | Good (29) |
| Redahan, 2013 (41) | Good | Fair | Good | Poor | Good | Very poor | Good | Poor | Good | Fair (26) |
| Sengupta, 2013 (42) | Poor | Good | Poor | Poor | Good | Very poor | Good | Poor | Poor | Fair (19) |
| Speer, 2013 (43) | Poor | Poor | Poor | Poor | Fair | Very poor | Good | Poor | Good | Fair (22) |
| Wallace, 2013 (44) | Good | Fair | Good | Poor | Good | Very poor | Good | Poor | Good | Good (28) |
| Weckmann, 2013 (45) | Poor | Poor | Poor | Poor | Good | Poor | Good | Poor | Fair | Fair (21) |
| Zdenkowski, 2013 (46) | Poor | Good | Fair | Poor | Good | Poor | Good | Poor | Good | Fair (27) |
| Zheng, 2013 (47) | Poor | Fair | Good | Poor | Good | Very poor | Good | Poor | Good | Fair (26) |
| Bogasky, 2014 (48) | Poor | Good | Fair | Poor | Good | Very poor | Good | Poor | Fair | Fair (25) |
| Brown, 2014 (49) | Good | Good | Good | Poor | Good | Poor | Good | Poor | Good | Good (30) |
| Casarett, 2014 (50) | Poor | Good | Good | Fair | Good | Very poor | Good | Good | Fair | Good (29) |
| Chai, 2014 (51) | Poor | Fair | Fair | Poor | Good | Good | Good | Poor | Fair | Fair (27) |
| Eastman, 2014 (52) | Poor | Good | Poor | Poor | Good | Very poor | Good | Poor | Fair | Fair (24) |
| Fullerton, 2014 (53) | Very poor | Poor | Very poor | Very poor | Very poor | Very poor | Poor | Poor | Poor | Poor (13) |
| Guay, 2014 (54) | Good | Fair | Poor | Poor | Good | Good | Good | Poor | Good | Good (29) |
| Hui, 2014 (55) | Fair | Good | Good | Poor | Good | Good | Good | Poor | Good | Good (31) |
| Kang, 2014 (56) | Poor | Poor | Good | Poor | Good | Poor | Good | Poor | Good | Fair (26) |
| Kao, 2014 (57) | Good | Good | Good | Poor | Good | Very poor | Good | Poor | Good | Good (29) |
| Keim-Malpass, 2014 (58) | Good | Fair | Fair | Poor | Good | Good | Good | Poor | Fair | Good (29) |
| Koivu, 2014 (59) | Good | Poor | Poor | Poor | Good | Very poor | Good | Poor | Poor | Fair (23) |
| Obermeyer, 2014 (60) | Good | Good | Poor | Poor | Fair | Poor | Good | Poor | Fair | Fair (26) |
| Olmsted, 2014 (61) | Poor | Good | Good | Poor | Good | Good | Good | Poor | Fair | Good (29) |
| Scheffey, 2014 (62) | Poor | Fair | Good | Poor | Good | Poor | Good | Poor | Poor | Fair (25) |
| Seow, 2014 (63) | Poor | Poor | Good | Poor | Good | Poor | Good | Poor | Good | Fair (26) |
| Sexauer, 2014 (64) | Good | Good | Poor | Poor | Good | Very poor | Good | Poor | Good | Fair (27) |
| Shin, 2014 (65) | Good | Poor | Fair | Poor | Good | Good | Good | Poor | Good | Good (29) |
| Unroe, 2014 (66) | Poor | Very poor | Very poor | Poor | Good | Very poor | Poor | Poor | Poor | Poor (17) |
| Wachterman, 2014 (67) | Good | Good | Good | Poor | Good | Very poor | Good | Poor | Good | Good (29) |
| Yamagishi, 2014 (68) | Good | Fair | Good | Poor | Good | Poor | Good | Poor | Good | Good (29) |
| Yeung, 2014 (69) | Poor | Fair | Good | Poor | Good | Good | Good | Poor | Fair | Good (28) |
| Alsirafy, 2015 (70) | Fair | Poor | Fair | Poor | Poor | Poor | Good | Poor | Fair | Fair (23) |
| Chiang, 2015 (71) | Good | Poor | Good | Poor | Good | Good | Good | Poor | Good | Good (30) |
| Choi, 2015 (72) | Poor | Poor | Good | Poor | Good | Poor | Good | Poor | Good | Fair (24) |
| Colman, 2015 (73) | Good | Very poor | Fair | Poor | Good | Poor | Good | Poor | Fair | Fair (25) |
| Dingfield, 2015 (74) | Good | Fair | Poor | Fair | Good | Poor | Good | Good | Good | Good (30) |
| Dougherty, 2015 (75) | Poor | Fair | Fair | Poor | Good | Poor | Good | Poor | Good | Fair (26) |
| El-Jawahri, 2015 (76) | Good | Fair | Poor | Poor | Good | Very poor | Good | Poor | Good | Fair (26) |
| Gage, 2015 (77) | Poor | Good | Good | Poor | Good | Good | Good | Poor | Good | Good (30) |
| Gozalo, 2015 (78) | Poor | Poor | Poor | Poor | Good | Very poor | Good | Poor | Fair | Fair (22) |
| Gu, 2015 (79) | Good | Good | Good | Poor | Good | Poor | Good | Poor | Fair | Good (29) |
| Gupte, 2015 (80) | Good | Good | Good | Poor | Good | Good | Good | Poor | Good | Good (32) |
| Hennemann-Krause, 2015 (81) | Poor | Good | Good | Poor | Good | Fair | Fair | Poor | Good | Good (28) |
| Hui, 2015 (82) | Good | Poor | Fair | Fair | Good | Good | Good | Good | Fair | Good (31) |
| Kao, 2015 (83) | Good | Good | Good | Poor | Good | Good | Good | Poor | Fair | Good (31) |
| Kim, 2015 (84) | Poor | Poor | Good | Poor | Good | Good | Good | Poor | Good | Good (28) |
| Kozlov, 2015 (85) | Poor | Poor | Poor | Poor | Poor | Very poor | Good | Poor | Good | Fair (21) |
| Lee, 2015 (86) | Fair | Poor | Good | Poor | Good | Very poor | Fair | Poor | Fair | Fair (24) |
| Myers, 2015 (87) | Fair | Poor | Poor | Poor | Fair | Very poor | Fair | Poor | Fair | Fair (21) |
| O’Connor, 2015 (88) | Good | Good | Fair | Poor | Good | Poor | Good | Poor | Good | Good (29) |
| Pineau, 2015 (89) | Very poor | Poor | Very poor | Very poor | Very poor | Very poor | Good | Very poor | Very poor | Poor (13) |
| Zakhour, 2015 (90) | Good | Good | Poor | Poor | Good | Poor | Good | Poor | Good | Good (28) |
| Bauman, 2016 (91) | Good | Poor | Good | Fair | Poor | Very poor | Good | Poor | Good | Fair (26) |
| Bennett, 2016 (10) | Good | Good | Fair | Poor | Good | Poor | Good | Poor | Good | Good (29) |
| Brooks, 2016 (92) | Good | Fair | Good | Fair | Good | Very poor | Good | Poor | Fair | Good (28) |
| Brown, 2016 (93) | Good | Good | Good | Poor | Good | Very poor | Good | Poor | Good | Good (29) |
| Cheraghlou, 2016 (94) | Good | Fair | Good | Fair | Poor | Poor | Good | Poor | Poor | Fair (26) |
| Diamond, 2016 (95) | Good | Poor | Good | Fair | Good | Fair | Good | Poor | Good | Good (30) |
| Hamano, 2016 (96) | Good | Fair | Good | Poor | Good | Poor | Good | Very poor | Poor | Fair (26) |
| Jarosek, 2016 (97) | Good | Good | Good | Fair | Good | Poor | Good | Poor | Good | Good (31) |
| Jegier, 2016 (98) | Good | Good | Good | Fair | Good | Poor | Good | Poor | Good | Good (31) |
| Kierner, 2016 (99) | Fair | Good | Good | Poor | Good | Very poor | Good | Poor | Fair | Fair (27) |
| King, 2016 (100) | Good | Good | Good | Poor | Good | Poor | Good | Poor | Fair | Good (29) |
| Lowe, 2016 (101) | Good | Fair | Good | Fair | Good | Poor | Good | Poor | Fair | Good (29) |
| Masman, 2016 (102) | Good | Fair | Good | Poor | Good | Poor | Good | Poor | Good | Good (29) |
| Obermeyer, 2016 (103)^111^ | Good | Poor | Poor | Poor | Good | Very poor | Good | Poor | Fair | Fair (24) |
| Odejide, 2016 (104) | Fair | Fair | Fair | Poor | Good | Very poor | Good | Poor | Good | Fair (26) |
| Perri, 2016 (105) | Good | Fair | Fair | Poor | Good | Poor | Good | Poor | Fair | Fair (27) |
| Porteous, 2016 (106) | Good | Fair | Poor | Poor | Good | Fair | Good | Poor | Good | Good (28) |
| Rosenwax, 2016 (107) | Good | Fair | Fair | Poor | Good | Fair | Good | Poor | Fair | Good (28) |
| Sathornviriyapong, 2016 (108) | Good | Good | Fair | Poor | Good | Fair | Good | Poor | Fair | Good (29) |
| Schmalz, 2016 (109) | Good | Fair | Poor | Poor | Good | Fair | Good | Poor | Fair | Fair (27) |
| Schur, 2016 (110) | Fair | Good | Fair | Poor | Good | Poor | Good | Poor | Fair | Fair (27) |
| Senderovich, 2016 (111) | Good | Fair | Fair | Fair | Good | Poor | Good | Good | Fair | Good (30) |
| Sharma, 2016 (112) | Good | Fair | Poor | Poor | Good | Poor | Good | Poor | Good | Fair (27) |
| Stevenson, 2016 (113) | Good | Good | Poor | Poor | Good | Very poor | Good | Poor | Good | Good (29) |
| United States Renal Data System, 2017 (114) | Poor | Poor | Poor | Poor | Fair | Very poor | Fair | Poor | Poor | Fair (19) |
| Adsersen, 2017 (115) | Good | Good | Fair | Poor | Good | Very poor | Good | Poor | Good | Good (28) |
| Chan, 2017 (116) | Very poor | Poor | Fair | Very poor | Good | Very poor | Fair | Poor | Fair | Fair (20) |
| Choi, 2017 (117) | Poor | Fair | Fair | Poor | Good | Poor | Good | Poor | Fair | Fair (25) |
| de la Cruz, 2017 (118) | Fair | Fair | Poor | Poor | Good | Poor | Good | Poor | Good | Fair (26) |
| Einstein, 2017 (119) | Good | Good | Poor | Poor | Good | Very poor | Good | Poor | Fair | Fair (26) |
| Forst, 2017 (120) | Fair | Good | Good | Poor | Good | Poor | Good | Poor | Fair | Good (28) |
| Fukui, 2017 (121) | Fair | Fair | Good | Poor | Good | Poor | Good | Poor | Fair | Fair (27) |
| Harris, 2017 (122) | Good | Fair | Fair | Poor | Good | Very poor | Good | Poor | Good | Fair (27) |
| Hoverman, 2017 (123) | Poor | Poor | Poor | Poor | Poor | Very poor | Good | Poor | Fair | Fair (20) |
| Kaufman, 2017 (124) | Poor | Fair | Fair | Poor | Good | Poor | Good | Poor | Good | Fair (26) |
| Kelly, 2017 (125) | Good | Fair | Good | Poor | Very poor | Fair | Good | Poor | Good | Fair (27) |
| Kuchinad, 2017 (126) | Good | Poor | Poor | Poor | Good | Fair | Good | Poor | Poor | Fair (25) |
| Lin, 2017 (127) | Good | Poor | Fair | Poor | Good | Poor | Good | Poor | Fair | Fair (26) |
| Lustbader, 2017 (128) | Poor | Poor | Poor | Poor | Good | Very poor | Good | Poor | Fair | Fair (22) |
| Masel, 2017 (129) | Good | Poor | Poor | Poor | Good | Poor | Good | Poor | Fair | Fair (25) |
| Mercadante, 2017 (130) | Good | Fair | Fair | Poor | Good | Fair | Good | Poor | Fair | Good (28) |
| O’Leary, 2017 (17) | Poor | Good | Good | Poor | Good | Good | Good | Poor | Fair | Good (29) |
| Otsuka, 2017 (131) | Good | Fair | Good | Poor | Good | Poor | Good | Poor | Poor | Fair (27) |
| Palmer, 2017 (132) | Poor | Fair | Fair | Poor | Good | Fair | Good | Poor | Good | Fair (27) |
| Pellizzari, 2017 (133) | Poor | Good | Good | Poor | Good | Good | Good | Poor | Fair | Good (29) |
| Rivet, 2017 (134) | Good | Good | Fair | Poor | Good | Fair | Good | Poor | Good | Good (30) |
| Sanoff, 2017 (135) | Fair | Poor | Good | Poor | Good | Very poor | Good | Poor | Fair | Fair (25) |
| Scaccabarozzi, 2017 (136) | Good | Poor | Fair | Poor | Good | Fair | Good | Poor | Fair | Fair (27) |
| Schuler, 2017 (137) | Fair | Good | Fair | Poor | Good | Fair | Good | Poor | Good | Good (29) |
| Senel, 2017 (138) | Good | Poor | Poor | Poor | Good | Good | Good | Poor | Fair | Fair (27) |
| Shah, 2017 (139) | Good | Fair | Fair | Poor | Good | Good | Good | Poor | Fair | Good (29) |
| Sharp, 2017 (140) | Good | Fair | Poor | Poor | Poor | Poor | Good | Poor | Poor | Fair (23) |
| Tanuseputro, 2017 (19) | Good | Fair | Good | Poor | Good | Poor | Good | Poor | Good | Good (29) |
| Taylor, 2017 (141) | Fair | Good | Fair | Poor | Good | Poor | Good | Poor | Fair | Fair (27) |
| Unroe, 2017 (142) | Fair | Fair | Good | Poor | Good | Poor | Good | Poor | Good | Good (28) |
| Vayne-Bossert, 2017 (143) | Fair | Good | Good | Poor | Good | Poor | Good | Poor | Good | Good (29) |
| Vinant, 2017 (144) | Good | Good | Fair | Poor | Good | Good | Good | Poor | Fair | Good (30) |
| Waite, 2017 (145) | Poor | Poor | Good | Poor | Poor | Poor | Good | Poor | Good | Fair (24) |
| Wang, Hsu, 2017 (146) | Fair | Good | Good | Poor | Good | Poor | Good | Poor | Good | Good (29) |
| Wang, Knight, 2017 (147) | Good | Good | Fair | Poor | Good | Poor | Good | Poor | Good | Good (29) |
| Wilson, 2017 (148) | Good | Good | Good | Poor | Good | Good | Good | Poor | Good | Good (32) |
| Yim, 2017 (149) | Good | Good | Good | Poor | Poor | Poor | Good | Poor | Fair | Fair (27) |
| Akdogan, 2018 (150) | Poor | Poor | Good | Poor | Good | Poor | Good | Poor | Good | Fair (26) |
| Allsop, 2018 (8) | Good | Good | Good | Poor | Good | Poor | Good | Poor | Good | Good (30) |
| Assareh, 2018 (151) | Good | Poor | Good | Poor | Good | Good | Good | Poor | Good | Good (30) |
| Cho, 2018 (152) | Poor | Fair | Fair | Poor | Good | Good | Good | Poor | Good | Good (28) |
| Choi, 2018 (153) | Good | Fair | Good | Poor | Good | Good | Good | Poor | Fair | Good (30) |
| de Oliveira Valentino, 2018 (154) | Good | Good | Fair | Fair | Good | Poor | Good | Fair | Fair | Good (30) |
| Dinҫer, 2018 (155) | Good | Poor | Good | Poor | Good | Poor | Good | Poor | Poor | Fair (26) |
| Duff, 2018 (156) | Good | Good | Fair | Poor | Good | Good | Good | Poor | Fair | Good (30) |
| Dunn. 2018 (157) | Poor | Poor | Good | Poor | Good | Very poor | Good | Poor | Very poor | Fair (22) |
| Gainza-Miranda, 2018 (158) | Good | Good | Good | Poor | Good | Poor | Good | Poor | Poor | Good (28) |
| Gidwani-Marszowski, 2018 (159) | Good | Good | Good | Poor | Good | Poor | Good | Poor | Good | Good (30) |
| Gill, 2018 (160) | Good | Poor | Good | Poor | Good | Good | Good | Poor | Fair | Good (29) |
| Gurau, 2018 (161) | Poor | Fair | Fair | Poor | Good | Poor | Good | Poor | Fair | Fair (25) |
| Hattori, 2018 (162) | Poor | Poor | Fair | Poor | Good | Good | Good | Poor | Fair | Fair (26) |
| Hausner, 2018 (163) | Poor | Fair | Fair | Poor | Good | Very poor | Good | Poor | Good | Fair (25) |
| Hung, 2018 (164) | Good | Good | Poor | Poor | Good | Very poor | Good | Poor | Fair | Fair (26) |
| Hutchinson, 2018 (165) | Fair | Good | Good | Poor | Good | Good | Good | Poor | Fair | Good (29) |
| Johnson, 2018 (166) | Good | Fair | Poor | Poor | Good | Very poor | Good | Poor | Good | Fair (26) |
| Kaufman, 2018 (167) | Poor | Good | Poor | Poor | Good | Very poor | Good | Poor | Fair | Fair (24) |
| LeBlanc, 2018 (168) | Fair | Fair | Fair | Poor | Good | Poor | Good | Poor | Good | Fair (27) |
| Ledoux, 2018 (169) | Good | Poor | Fair | Poor | Good | Poor | Good | Poor | Good | Fair (27) |
| Lo, 2018 (170) | Good | Fair | Fair | Poor | Good | Poor | Good | Poor | Fair | Fair (27) |
| McDermott, 2018 (171) | Fair | Fair | Fair | Poor | Good | Poor | Good | Poor | Good | Fair (27) |
| Mendieta, 2018 (172) | Poor | Fair | Poor | Poor | Good | Very poor | Good | Poor | Fair | Fair (23) |
| Merchant, 2018 (173) | Fair | Fair | Good | Poor | Good | Poor | Good | Poor | Poor | Fair (26) |
| Mulville, 2018 (174) | Good | Poor | Very poor | Poor | Poor | Very poor | Very poor | Poor | Poor | Poor (17) |
| Nazim, 2018 (175) | Poor | Fair | Good | Poor | Good | Poor | Good | Poor | Fair | Fair (26) |
| O'Hare, 2018 (176) | Fair | Poor | Fair | Poor | Fair | Very poor | Good | Poor | Good | Fair (24) |
| Rozman, 2018 (177) | Fair | Good | Fair | Poor | Good | Very poor | Good | Poor | Fair | Fair (26) |
| Shih, 2018 (178) | Fair | Poor | Poor | Poor | Good | Good | Good | Poor | Fair | Fair (26) |
| Shinall Jr, 2018 (179) | Poor | Fair | Fair | Poor | Good | Good | Good | Poor | Fair | Fair (27) |
| Stephens, 2018 (180) | Good | Fair | Fair | Poor | Good | Poor | Good | Poor | Good | Good (28) |
| Vogl, 2018 (181) | Fair | Good | Poor | Poor | Good | Poor | Good | Poor | Good | Fair (27) |
| Wadhwa, 2018 (182) | Good | Good | Poor | Poor | Good | Poor | Good | Poor | Good | Good (28) |
| Yennurajalingam, 2018 (183) | Fair | Fair | Good | Poor | Good | Poor | Good | Poor | Fair | Fair (27) |
| Yoo, 2018 (184) | Good | Fair | Good | Poor | Good | Good | Good | Poor | Good | Good (31) |
| Ziegler, 2018 (185) | Good | Good | Fair | Poor | Good | Poor | Good | Poor | Good | Good (29) |

Rating for individual scores: Good = 4, Fair = 3, Poor = 2, Very poor = 1

**Table S4: Summary of studies characteristics (excluding USA data).**

|  | | **No. of studies (%)** | **No. of patients (%)** |
| --- | --- | --- | --- |
| **Total number** | | 83 (100%) | 397,118 (100%) |
| **UNDP Human Development Index (2015)** | Very high | 74 (89·2%) | 391,995 (98·7%) |
|  | High | 8 (9·6%) | 5,000 (1·3%) |
|  | Medium | 1 (1·2%) | 123 (0·0%) |
| **WPCA categorisation of palliative care development (2011)** | 4b | 55 (66·3%) | 234,823 (59·1%) |
|  | 4a | 13 (15·7%) | 152,262 (38·3%) |
|  | 3b | 3 (3·6%) | 603 (0·2%) |
|  | 3a | 12 (14·5%) | 9,430 (2·4%) |
| **Type of disease** | Malignant | 45 (54·2%) | 198,328 (49·9%) |
|  | Non-malignant | 10 (12·0%) | 823 (0·2%) |
|  | Mixed | 24 (28·9%) | 191,209 (48·1%) |
|  | Unspecified | 4 (4·8%) | 6,758 (1·7%) |
| **Type of palliative care service** | Specialist palliative care unit | 31 (37·3%) | 20,019 (5·0%) |
|  | Community/home | 12 (14·5%) | 8,643 (2·2%) |
|  | General hospital ward | 5 (6·0%) | 26,609 (6·7%) |
|  | Combined | 33 (39·8%) | 341,725 (86·0%) |
|  | Unspecified | 2 (2·4%) | 122 (0·0%) |
| **Level of analysis** | Local | 51 (61·4%) | 25,626 (6·5%) |
|  | Regional | 18 (21·7%) | 174,516 (43·9%) |
|  | National | 14 (16·9%) | 196,976 (49·6%) |
| **Statistical summary used to report duration of care** | Solely mean | 30 (36·1%) | 140,147 (35·3%) |
|  | Solely median | 39 (47·0%) | 102,715 (25·9%) |
|  | Median and mean | 14 (16·9%) | 154,256 (38·8%) |
| **Terminology used to report duration of care** | Referral-to-death | 41 (49·4%) | 360,905 (90·9%) |
|  | Survival | 13 (15·7%) | 13,155 (3·3%) |
|  | Length-of-stay | 29 (34·9%) | 23,058 (5·8%) |
| **No. study participants** | <100 | 20 (24·1%) | 1,083 (0·3%) |
|  | ≥100 | 63 (75·9%) | 396,035 (99·7%) |
| **Participants alive at the end of the study period** | ≤5% | 56 (67·5%) | 375,572 (94·6%) |
|  | >5% | 27 (32·5%) | 21,546 (5·4%) |
| **Hawker’s criteria rating** | Poor | 2 (2·4%) | 66 (0·0%) |
|  | Fair | 44 (53·0%) | 187,152 (47·1%) |
|  | Good | 36 (43·4%) | 209,819 (52·8%) |
|  | Missing* | 1 (1·2%) | 81 (0·0%) |

**Table S5. Duration of care with sub-analyses (excluding USA data).**

|  | | **Weighted median duration of care in days (IQR)** | **p value** |
| --- | --- | --- | --- |
| **Total number** | | 29·00 (40·88) |  |
| **UNDP Human Development Index (2015)** | Very high | 29·00 (40·88) |  |
|  | High | 34·00 (0) |  |
|  | Medium | 66·00 (*†) |  |
|  | Very high | 29·00 (40·88) | p<0·001 |
|  | < Very high | 34·00 (0) |  |
| **WPCA categorisation of palliative care development (2011)** | 4b | 68·88 (34·88) |  |
|  | 4a | 28·00 (0) |  |
|  | 3b | 24·88 (12·35) |  |
|  | 3a | 22·18 (11·82) |  |
|  | 4b | 68·88 (34·88) | p<0·001 |
|  | < 4b | 28·00 (0) |  |
| **Type of disease** | Malignant | 28·00 (1·00) | p<0·001 |
|  | Non-malignant | 24·27 (109·00) |  |
|  | Mixed | 48·00 (34·88) |  |
|  | Unspecified | 14·81 (0) |  |
| **Type of palliative care service** | Specialist palliative care unit | 14·81 (7·37) | p<0·001 |
|  | Community/home | 47·92 (44·08) |  |
|  | General hospital ward | 6·00 (0) |  |
|  | Combined | 34·00 (40·88) |  |
|  | Unspecified | 179·40 (167·40) |  |

IQR = interquartile range; UNDP = United Nations Development Programme; * = data neither available nor possible to calculate; † = IQR from single study sample; WHPCA = Worldwide Hospice Palliative Care Alliance
